# Forecasting the 2017/2018 seasonal influenza epidemic in England using multiple dynamic transmission models: a case study

**DOI:** 10.1186/s12889-020-8455-9

**Published:** 2020-04-15

**Authors:** Paul J. Birrell, Xu-Sheng Zhang, Alice Corbella, Edwin van Leeuwen, Nikolaos Panagiotopoulos, Katja Hoschler, Alex J. Elliot, Maryia McGee, Simon de Lusignan, Anne M. Presanis, Marc Baguelin, Maria Zambon, André Charlett, Richard G. Pebody, Daniela De Angelis

**Affiliations:** 1 MRC Biostatistics Unit, University of Cambridge, Cambridge Institute of Public Health, Robinson Way, Cambridge, CB2 0SR UK; 2 National Infection Service, Public Health England, 61 Colindale Avenue, London, NW9 5EQ UK; 3 Virus Reference Department, National Infection Service, Public Health England, 61 Colindale Avenue, London, NW9 5EQ UK; 4 Real-time Syndromic Surveillance Team, Field Service, National Infection Service, Public Health England, 5 St Philip’s Place, Birmingham, B3 2PW UK; 5 Nuffield Department of Primary Care Health Sciences, University of Oxford, Oxford, OX2 6GG UK; 6 Royal College of General Practitioners Research and Surveillance Centre, 30 Euston Square, London, NW1 2FB UK

**Keywords:** Transmission models, Seasonal influenza, Intensive care admissions, GP consultations, Nowcasting, Forecasting

## Abstract

**Background:**

Since the 2009 A/H1N1 pandemic, Public Health England have developed a suite of real-time statistical models utilising enhanced pandemic surveillance data to nowcast and forecast a future pandemic. Their ability to track seasonal influenza and predict heightened winter healthcare burden in the light of high activity in Australia in 2017 was untested.

**Methods:**

Four transmission models were used in forecasting the 2017/2018 seasonal influenza epidemic in England: a stratified primary care model using daily, region-specific, counts and virological swab positivity of influenza-like illness consultations in general practice (GP); a strain-specific (SS) model using weekly, national GP ILI and virological data; an intensive care model (ICU) using reports of ICU influenza admissions; and a synthesis model that included all data sources. For the first 12 weeks of 2018, each model was applied to the latest data to provide estimates of epidemic parameters and short-term influenza forecasts. The added value of pre-season population susceptibility data was explored.

**Results:**

The combined results provided valuable nowcasts of the state of the epidemic. Short-term predictions of burden on primary and secondary health services were initially highly variable before reaching consensus beyond the observed peaks in activity between weeks 3–4 of 2018. Estimates for *R*_0_ were consistent over time for three of the four models until week 12 of 2018, and there was consistency in the estimation of *R*_0_ across the SPC and SS models, and in the ICU attack rates estimated by the ICU and the synthesis model. Estimation and predictions varied according to the assumed levels of pre-season immunity.

**Conclusions:**

This exercise successfully applied a range of pandemic models to seasonal influenza. Forecasting early in the season remains challenging but represents a crucially important activity to inform planning. Improved knowledge of pre-existing levels of immunity would be valuable.

## Background

The evolution of influenza viruses results in annual epidemics of seasonal influenza, with less frequent global pandemics occurring due to the emergence of novel influenza viruses to which there is little population immunity. Both have the potential to cause substantial burden of disease to the population and to present significant challenges to already strained healthcare systems [1–3]. Many countries have invested substantial resources to developing epidemiological and virological surveillance tools to rapidly detect the onset of the influenza season each year [4, 5]; to measure the level of activity and the impact on the health service; and to characterise the main circulating virus strains and how well they match the seasonal vaccine. Attempts, however, to undertake seasonal influenza forecasting have been historically challenging due to the complex interplay between the influenza virus, population immunity, and environmental factors [6]. Nonetheless, progress has been made in the provision of short-term predictions of influenza in some countries in recent years particularly in the United States [5, 7], encouraging similar endeavours elsewhere.

In the UK, the National Risk Register of Civil Emergencies lists an outbreak of pandemic influenza as the greatest risk faced by its population [8]. Before, during and after 2009 A/H1N1pdm, quantitative approaches for real-time modelling and forecasting burden have been developed [9–11]. The availability of these models, together with complementary surveillance and data collection systems including sero-epidemiology for seasonal influenza, provided the opportunity to address the challenge of predicting seasonal influenza activity in England. This became a pressing need, when, following a particularly intense 2017 influenza season in Australia [12], prior to the winter season 2017/8 the National Health Service (NHS) put in place winter preparedness plans to manage potential acute pressures on the health service [13]. Ultimately, England experienced the most intense influenza season since the first post-pandemic season in 2010/11 [13]. Questions were raised about how the epidemic would evolve, when it would peak, how intense the peak in activity would be, and what would be the resulting demands on the health service, in terms of burden on GPs, hospitals, and intensive care units.

This study reports the advanced analytical and modelling experience at a national level led by Public Health England (PHE) during the 2017–2018 influenza season and in particular the attempt to undertake short- and medium-term forecasts of influenza activity and the impact of influenza on the health care service.

## Methods

### Data

The annual monitoring of influenza activity in England is based on a series of data streams. Those specifically utilised for the purposes of this work were weekly and daily consultations for influenza-like illness (ILI) in general practice (GP); virological testing of respiratory swabs obtained from patients consulting in GP; laboratory confirmed influenza admissions to hospitals and intensive care and high dependence units (ICU/HDU); and population-based strain- and age-specific serological data on influenza immunity. These data sources have been described in detail elsewhere and we review them only briefly here [14–17].

The GP ILI data represents patients attending primary care with acute ILI, a proportion of which will be due to influenza infection. Data were obtained from two sources: the Weekly Returns Service of the Royal College of General Practitioners (RCGP) Research and Surveillance Centre (RSC), a sentinel GP network covering a weekly population (in December 2017) of approximately 2 million people from over 200 practices [18]; and the PHE GP in-hours-syndromic-surveillance system, which collates daily ILI consultations stratified by, amongst other things, age group and NHS region from approximately 3,500 GP practices, representing about 50% of the total practices in England [16]. Both of these data sources are routinely available throughout the year, with a period of enhanced influenza surveillance starting at week 40, and ending at week 20, irrespective of the amount of circulating influenza. The RCGP RSC sentinel network has integrated virological monitoring [18], whereby GPs undertake respiratory swabbing of a subset of patients consulting for ILI. Swabs are tested by reverse transcription-polymerase chain reaction to identify the component of ILI due to each influenza strain through the presence of type and subtype specific positive swabs in the sample.

Data on influenza confirmed hospitalizations and intensive care admissions are collected through the UK Severe Influenza Surveillance System (USISS) [15, 19]. Weekly numbers of laboratory-confirmed influenza cases (of all the commonly circulating strains: A/H1N1pdm09, A/H3N2, B) admitted to an ICU or high dependence unit (HDU) and the number of confirmed influenza deaths in ICU/HDU are reported from all NHS trusts in England from week 40 to week 20 of the following year, alongside attendant information including age and influenza subtype. In addition to this mandatory scheme, a subgroup of NHS trusts in England is recruited every year to participate in the USISS sentinel scheme [20], which provides weekly numbers of laboratory confirmed influenza hospitalisations (A/H1, A/H3 and B).

A final source of information is provided by intra-seasonal cross-sectional population-based serological survey data. These data provide a measure of the seroprevalence of strain-specific antibodies to A/H3, A/H1 and B influenza viruses using haemagglutination inhibition (HAI) assays with an HAI titre >1/40. These data inform the susceptibility of the population after the end of the previous season, but prior to both the seasonal influenza vaccination campaign (which should boost population immunity) and the onset of seasonal virus circulation.

### Influenza transmission models

Four deterministic compartmental transmission models were employed to estimate and forecast the evolution of the 2017/18 seasonal influenza in England: a Stratified Primary Care Surveillance Model (SPC); a Strain-Specific model (SS); a Severity-based model (ICU); and a Synthesis model. The common transmission structure is of the Susceptible (S), Infectious (I), Removed (R) type, adapted for greater realism to include additional Exposed (E) or I compartments in some cases. All the models assume homogeneous mixing between susceptible and infected individuals within population strata. Transmission dynamics are linked to the observed data through appropriate disease reporting models. The data streams used to estimate model parameters and relevant epidemic quantities vary according to the model. Detailed information on the models’ structure, data used, distributional assumptions and estimation approaches is given in the Web Extra Mate-rial(Sections A–B; Table S1) while here we give a short summary of each.

**Stratified Primary Care Model** This is a SEEIIR model, a modified version of the model developed to reconstruct the 2009 H1N1 influenza pandemic [11, 21, 22]. The model uses daily (or weekly) data on the number of GP consultations for ILI from the PHE influenza surveillance dataset augmented with the RCGP’s virological data to estimate the component of ILI due to influenza. In the first week of analysis, the number of ILI consultations due to influenza were simply estimated by multiplying the total number of consultations in sentinel RCGP practices by the proportion of swabs testing positive for an influenza virus, obtaining what we term ILI+. When more detailed data became available, it was possible to model jointly daily ILI counts and swab positivity data, appropriately accounting for the size of the virological samples, and to specify distinct epidemic models in each of five regions in England [22]. A definition of the regions used is available in the Web Extra Material (Section B.1.1). When using ILI rather than ILI+, it is necessary to account for "background" rates of consultation, the component of ILI not attributable to influenza. This level of consultation is estimated by fitting an endemic/epidemic model [23] to 3 years of historic ILI data prior to the 2017/2018 flu season.

**Strain-Specific Model** The SS model has an SIR structure for each of the three influenza strains (A/H1, A/H3 and B), which are assumed to transmit independently within the population [24]. The model uses weekly GP consultations for ILI from the RCGP RSC, together with the strain-specific virological data from the RCGP RSC’s virological monitoring to identify the contribution of each strain to the overall number of consultations. Serological data were used to inform strain-specific susceptibility at the start of the season.

**ICU-based Model** The ICU model for seasonal influenza represents a development of an existing influenza SEEIIR transmission model, which uses intensive care influenza admissions from the USISS system [25]. The transmission rate is allowed to vary over time to account for school holiday periods and the possible effects of changing patterns of interaction between age groups [26]. Transmission dynamics are linked to the observed ICU admissions through a delay from infection to admission and through the assumption that only a proportion of the infections is admitted to ICU.

**Synthesis Model** The synthesis model uses three data streams to estimate the underlying transmission dynamics: ICU and hospital admissions from USISS and the ILI+ dataset derived from RCGP surveillance. Transmission dynamics are described by a SEEIIR model assuming a constant transmission rate and random mixing. The basic feature of the disease reporting process are taken from previous studies [15, 21, 25].

### Estimation of parameters and quantities of interest

Using weeks as defined by the International Organisation for Standardization (ISO), the above models were run weekly in each of weeks 1 to 12 of 2018, with all analyses covering the period from week 40 of 2017 (denoted 2017w40), which started on the 2^nd^ October. For each week and each model, we estimate model parameters (see Table S1 in the Web Extra Material) in a Bayesian framework. Quantities that can be estimated from all models and have a common interpretation were the focus of comparisons. In particular, we estimated features of the epidemic that are of great public health interest, including the timing and magnitude of the peak in the burden on healthcare services (influenza-related visits to GPs, hospital and ICU admissions); *R*_*e*_(*t*), the effective reproduction number, representing the average number of infections generated by a single infection in the population (see Web Extra Material); and the propensity of infected cases to interact with the particular healthcare services (GP, Hospital, ICU). In deterministic models, the reproduction number decays over time with the depletion of susceptibles, so estimates of its value at the start of the influenza season only are presented and this quantity is denoted *R*_*e*_≡*R*_*e*_(0).

### Forecasting

From the estimates above it is possible to derive estimates of burden in terms of new infections, GP consultations and admissions to secondary care over the coming weeks. However, these estimates refer to quantities that are not directly observable from available data. Even GP consultations and data on admissions for severe disease typically refer only to a sub-sample of the whole population, representing a noisy version of these true underlying quantities. To assess the ability of the models to make predictions, it is necessary to contrast the model-based forecasts for observable quantities with the corresponding subsequent observations. We carried out such assessment by constructing posterior predictive distributions for future data points and plotting them alongside the observed values [7, 27].

## Results

### Parameter estimation

**Fig. 1:**
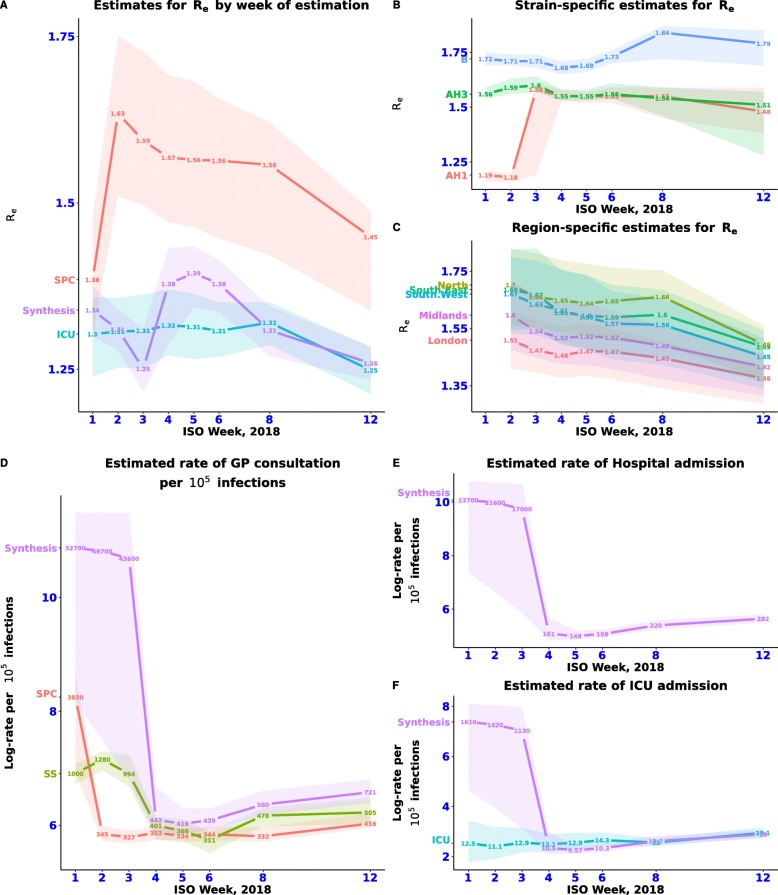
Evolution of estimates of key parameters over time. Estimates of effective reproductive number *R*_*e*_ (panels **a**–**c**) and of the log of the rates of GP consultation and admission to hospital and ICU over time per 100,000 infections (panels **d**–**f**). In panels **d**–**f** estimates are only plotted for models that included the relevant data

Detailed information on estimates from all models including uncertainty can be found in Table S2 in the Web ExtraMaterial. Here we compare estimates (and 95% Credible Intervals) of common parameters from the different modelling approaches as they are estimated over successive weeks. Note that as not all models provide comparable outputs, each panel of Fig. 1 refers only to the relevant models. Panel A reports estimates of the overall effective reproductive number *R*_*e*_ from three of the models (SPC, ICU and Synthesis), whereas Panel B refers to the strain-specific *R*_*e*_ obtained from the SS model. The SPC model persistently estimates the highest *R*_*e*_ in Panel A, with the central estimate comparable to the strain-specific estimates in Panel B. Estimates from the Synthesis and ICU models seem also to be consistent, converging to very similar values (1.25–1.26) by ISO week 12 (Panel C). Only the SS model made use of the population intra-seasonal seroprevalence data.

Panels D–F report estimates of health care seeking rates per 100,000 infections, of GP consultation for ILI (Panel D), hospitalisation (Panel E) and ICU admission (Panel F). These rates are not estimated if a model doesn’t utilise the relevant dataset. In Panel D, after an initial volatile pattern, the estimates settle down around 2018w4. In the SPC model, the ILI+ data used in 2018w1 are replaced from 2018w2 by separate GP ILI and swab positivity datasets. As a result, the estimated rate of GP consultation drops sharply and becomes much more precise. For the SS and SPC models, estimates of the propensity to consult in GP stabilise, over time, at around 0.5%. Results from the Synthesis model of all three healthcare seeking events, suggest a slightly higher value for the GP consulting rate, a figure of around 0.3% of infections leading to hospitalisation, with a lower estimate, below 0.02% of infections requiring ICU admission, in agreement with the ICU model whose estimates are the most consistent over the period.

### Estimating timing and level of peak activity in primary and secondary care

**Fig. 2:**
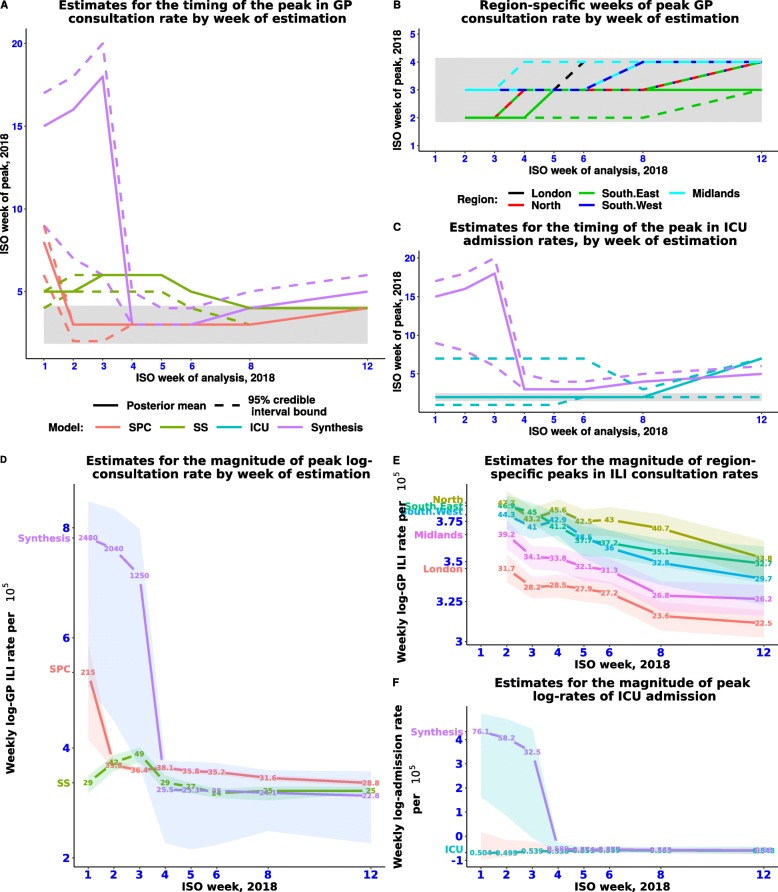
Evolution of forecasts of peak activity over time. Estimates for the peak timing (Panels **a**–**c**) and magnitude (Panels **d**–**f**) in GP consultations and ICU admissions over time. In Panels **a**–**c** the shaded grey region gives the time interval over which the observed peaks in ILI (and ILI+, Panels **a** and **b**) and ICU admission (Panel **c**) occur. In places, the credible intervals vanish, where the peak week is predicted with certainty to be in a specific week. Again, region-specific estimates are presented in panels **b** and **e**, while only the ICU and Synthesis models forecast the ICU admission rates and only these models feature in Panels **c** and **f**

Figure 2 displays estimates (posterior means), with uncertainty (95% Credible Intervals) of the timing of the peak and the peak intensity for the influenza-attributable ILI consultation rates and ICU admission rates by model and analysis time. The grey-shaded band on each plot gives the period of time over which peaks in the datasets were observed. Specifically, Panel A shows the evolution of the estimate of the peak week in GP ILI consultations as data accumulate, from some initial, quite heterogeneous, estimates, to a consensus estimate. For the GP consultation rates this consensus, placing the peak between 2018w3 and 2018w8, is only reached after 2018w4. Before that the uncertainty attached to the initial estimates varies substantially by model: the Synthesis model provides volatile results, while both the SPC and SS models are giving by this time a more precise indication on the position of the peak. The SPC model’s estimates seem overly precise, perhaps due to a lack of uncertainty in the estimation of the non-influenza ILI consultation rates (see Section B.1 in the Web Extra Material). Note also that the SPC model is able to provide region-specific estimates (Panel B). A similar message comes from the estimation of peak in the ICU rates, with an initial uncertainty diminishing after 2018w4, once a peak in the ICU data has definitely occurred.

In estimating the magnitude of the peak in GP consultations (Panel D), both the Synthesis model and the SPC model give initially a large estimate (based on ILI+ data). The SPC model prediction drops quickly in 2018w2, whereas the Synthesis model takes two additional weeks to do so. Over time the magnitude of the peak GP activity estimated under each of the three models gradually declines, with the SPC model continuing to estimate slightly higher peak activity. Although more consistent over time, the estimates for the peak intensity in the SS and SPC models again seem to be very precise given the predicted fall in activity over time. In Panel E, the regional estimates for the peak intensity behave in much the same way as the regional estimates for *R*_*e*_ with a gradual decline over time, with London and the Midlands having the lowest rates of peak activity and the North and South-East the highest. Estimates for the peak levels of ICU activity (Panel F) from the ICU model are very consistent over time, with the estimates from the Synthesis model converging to similar values from 2018w4 onwards. Table 1 presents the forecasts for the attack rates, the cumulative incidence of GP consultation, hospital and ICU admission over the course of the influenza season. These forecasts behave similarly to the forecasts for peak incidence (see Fig. 2d–f), in that they take a similar amount of time to converge to stable values. However, over time, the attack rate forecasts by the ICU model are steadily increasing (due to a slower than anticipated decline in the number of admissions), whereas those for the SS model are decreasing. There is little overlap between credible intervals from week 5 to week 12 across all models except the Synthesis model where forecasts are significantly less certain. Forecast ILI+ attack rates from the SS model tend to be higher than those from the SPC and Synthesis models, whereas forecasts from attack rates of ICU admission are comparable between the ICU and Synthesis models. The Synthesis model does, however, appear to estimate a high ratio between the number of hospital admissions and GP consultations, with there being less than three GP consultations per Hospitalisation, suggesting some possible unaccounted-for bias in the data sources.

**Table 1:** Forecast attack rates

ISO	SPC model	SS model	ICU model	Synthesis model		
Week	ILI+	ILI+	ICU	ILI+	Hosp.	ICU
1	1650	365	4.54	19400	8730	594
	(456, 2660)	(315, 424)	(2.36,10.8)	(163, 40700)	(738, 19200)	(50.1, 1320)
2	188	485	4.10	18500	8020	528
	(175, 206)	(415, 549)	(2.73, 8.13)	(1580, 33700)	(710, 15200)	(46.6, 1010)
3	174	570	4.74	8460	3320	220
	(165, 183)	(473, 648)	(3.57, 7.80)	(134, 20600)	(74.1, 8270)	(4.88, 555)
4	185	370	4.53	197	71.7	4.63
	(176, 194)	(322, 417)	(3.76, 6.04)	(61.1, 441)	(55.8, 128)	(3.54, 8.49)
5	175	348	4.80	190	67.4	4.35
	(167, 184)	(306, 378)	(4.11, 6.15)	(68.0, 331)	(57.2, 82.1)	(3.64, 5.42)
6	180	300	5.29	195	70.6	4.56
	(171, 189)	(262, 345)	(4.63, 6.40)	(71.4, 329)	(61.0, 83.1)	(3.89, 5.47)
8	173	280	5.01	225	85.3	5.29
	(164, 182)	(236, 305)	(4.68, 5.44)	(110, 354)	(70.9, 104)	(4.38, 6.53)
12	194	265	6.16	250	96.3	6.13
	(185, 202)	(249, 302)	(5.50, 6.97)	(108, 400)	(81.0, 115)	(5.10, 7.42)

Posterior median (with 95% credible intervals underneath) for the ILI+ attack rate (per 100,000 people), Hospitalization attack rate (per 100,000 people), and ICU attack rate (per 100,000 people). These attack rates are forecasts for the cumulative total number of GP consultations/Hospitalisation/ICU admissions attributable to influenza over the course of the whole influenza season. The ‘ISO Week’ gives the week of 2018 in which the forecast is made

### Forecasting activity and impact including timing of the peak

**Fig. 3:**
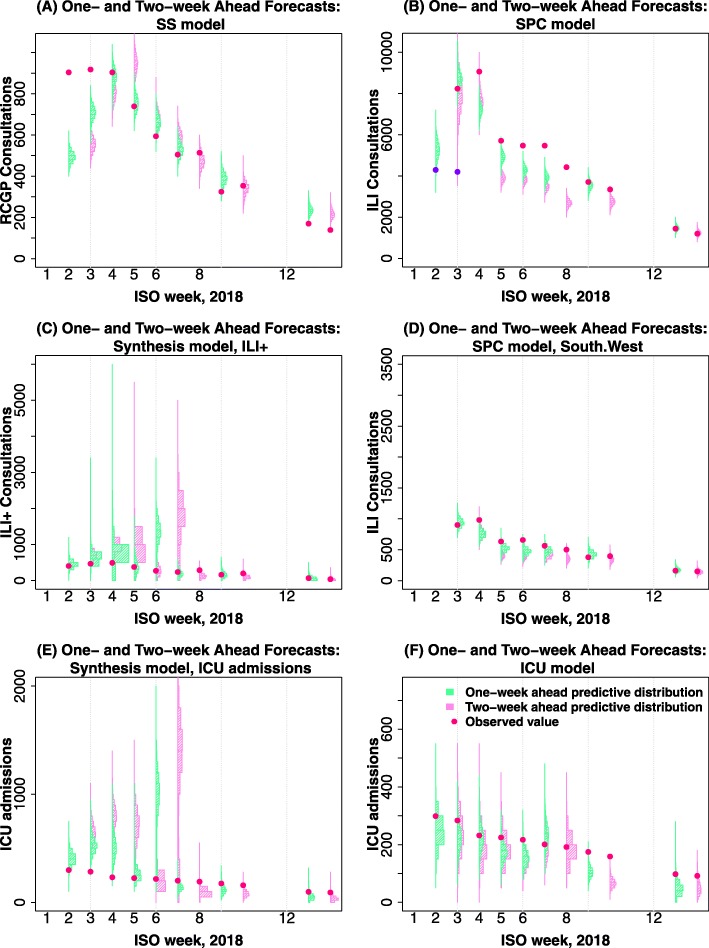
One- and Two-week ahead forecasting performance of the models Credible intervals for one week ahead (green) and two week ahead (pink) forecasts for the data used by each model. The red dots represent observed data, with blue dots in Panel **b** giving observed ILI+ observations (which were not used for model fitting beyond 2018w1)

Moving from estimation to forecasting, Fig. 3 illustrates the ability of each model to forecast relevant quantities. Here, at each time in each plot, one-week ahead probabilistic forecasts made in the previous week (in green) and two-week ahead forecasts made two weeks prior (in pink) are plotted alongside the data point subsequently observed at that time (red dots, with blue dots in Panel B corresponding to the ILI+ data that the SPC model used in week 1). Panel A displays the GP ILI forecasts from the SS model, showing how the model struggles to anticipate the peak in observed consultations early in 2018 and how the forecasts improve over 2018w5–9, after peaks in activity have been observed. Forecasts from the SPC model (Panel B) show a different pattern, displaying a better forecasting ability in the early period, which degrades over 2018w5–9, before very accurately predicting the GP ILI activity in 2018w12. This lack of forecasting ability over 2018w5–9, however, is due to the overly precise estimation observed above. Across each of the individual regions, the observed ILI consultations over 2018w5–9 fall in the upper tail of the posterior predictive distributions (as exemplified in Panel D for the South West), but they combine to give a national total ILI consultation that lies comfortably above the predictive distribution as seen in Panel B. The Synthesis model forecasts for the ILI data are shown in Panel C. The one week-ahead forecasts appear to perform reasonably well, but there is often high uncertainty attached to the two-week ahead forecasts, at times at odds with the one-week ahead forecast produced the following week. A similar big uncertainty is observed when using the Synthesis model to forecast ICU admissions (Panel E). Finally, the ICU model seems to exhibit good one-week and two-week forecasting performance, with almost all the data points lying within appropriate predictive intervals. However, it is to be noted that none of the observed values lie in the lower tail of the predictive distributions, suggesting some likely under-estimation of the ICU admissions over time.

## Discussion

This study shows how models designed for pandemic influenza could be adapted to answer questions on unfolding seasonal flu activity, in particular when it will peak, what the level of peak activity will be and what is the health service impact in terms of hospital and ICU admissions. We addressed these questions through a number of available transmission models, each sharing a common population compartment structure, that used a range of different standard data streams to make sequential estimation of disease spread, of case/severity indicators, and of future epidemic activity, all as data accumulated over time. Estimates were typically consistent across models using similar information (e.g. the SPC and SS models) and generally more stable towards the latter stages of the season. There is a high-degree of volatility in estimates that rely on the ILI+ data (Synthesis model and SPC model in week 1). This is most likely due to the ILI+ data being reliant on virological swab positivity data that only has a relatively small number of positives. These data then represent a highly noisy signal of flu activity that could easily conflict with the other datasets used by the Synthesis model. Estimation of timing and magnitude of peak activity was particularly challenging, as heterogeneous estimates were obtained from the different models in the early stages prior to observing peaks in the data.

To understand the predictive ability of the models, one– and two–week ahead forecasts were produced from each model and contrasted with the corresponding quantity subsequently observed. In this regard, models using a single data stream were typically more reliable, with prediction performance improving over time. More formal methods for assessing the forecast ability of a model exist, which properly account for both coverage and precision of the forecasts [27]. While interesting to investigate, these are more appropriate to situations when comparing different models forecasting the same indicators, unlike here. Informally assessed, the SPC model which incorporated daily surveillance data was best able to forecast peak activity, highlighting the importance of daily data streams for nowcasting and forecasting purposes.

Each of the models used have some shared characteristics and consequently some shared limitations. They each have at their heart deterministic transmission structures relying on assumptions of heterogeneous mixing (in the SPC model this heterogeneity is within regions, not England-wide). Deterministic dynamics are suitable for capturing pandemic dynamics when there is a single circulating influenza strain to which the population is almost entirely susceptible. With seasonal influenza both environmental and demographic stochastic effects will be influential, particularly in absorbing any lack of fit. Here, for example, the single-strain models struggled to adapt to the patterns in the data that are not characteristic of SEIR-type dynamics and in particular the slow decline of influenza activity following the peak. This lead to increased estimates of attack rates in 2018w12. Conversely, the deterministic dynamics of the strain-specific model couldn’t account for the presence of low-levels of influenza A/H1 activity without eventually leading to more widespread transmission. The increased flexibility in stochastic transmission models could potentially absorb this lack of fit without biasing estimates to the same extent.

A sensitivity analysis was conducted to understand the role that serological data can have in the monitoring of seasonal influenza (see Web Extra Material). The initial susceptibility of a population, which is a parameter in the models used here, cannot be estimated through the use of the surveillance streams alone. External knowledge on levels of population immunity prior to the start of the season needs to be provided, knowledge to which estimates of infection spread, attack rates, and case/severity indicators are found to be highly sensitive. The sensitivity study showed that while *R*_*e*_ estimates are robust to the levels of initial susceptibility, estimates for the transmissibility (measured by *R*_0_, see Web Extra Material) are inversely proportional to the susceptibility. Assuming higher initial susceptibility, the unaffected estimates of *R*_*e*_ will lead to higher rates of infection, as has been shown elsewhere [28]. To be able to explain the observed data there is a consequent impact on the case/severity ratios, which are estimated to be lower, leading here to differing estimates of (earlier) timing and magnitude of (higher) peak activity if early in the season. To weaken our strong assumption of homogeneous mixing, serological data can be of even further value if they are sufficiently representative (by age, strain, region etc.) to permit corresponding stratification of the transmission model. Additionally, the contribution of influenza vaccine programmes (in terms of uptake and effectiveness) to susceptibility also needs to be taken into account, as the vaccine campaign occurs after the intra-seasonal sero-survey is completed and will clearly further affect the population sero-profile prior to the start of the season. This will have been of particular importance in 2017/18, due to the apparent impact of vaccine-related egg-adaption, which may have reduced vaccine effectiveness against the circulating influenza A(H3N2) strains [29]. Further work on the potential role of sero-epidemiology to improving forecasting needs to be explored [30, 31] and a pilot study is ongoing [32].

## Conclusions

This exercise constituted a first attempt at establishing routine short-term forecasting of seasonal influenza activity in the UK with the aim of informing health service planning during the winter. The UK has a strong, integrated influenza surveillance system which provides an important opportunity to develop such approaches. Although modelling in the earlier stage of the season remains particularly challenging this has been a very valuable enterprise, identifying the key information requirements and the optimal modelling approaches. Reliable short-term predictions, particularly at local levels, for the number of cases in primary and secondary care can enable health service planners to optimally deploy limited capacity (e.g. hospital bed management). The work described here has identified the further developments required to achieve this: the use of more detailed serological data, the incorporation of information on vaccine coverage and building in additional flexibility to models to give less precise forecasts due to the presence of possible biases.

## Supplementary information

**Additional file 1** Web extra material. Appendix to this manuscript containing technical details of each of the models discussed here, some additional results and a sensitivity study on the impact of serological information.

## Data Availability

All datasets used are publicly available. Requests for access should be directed to: Richard.Pebody@phe.gov.uk. Computer code used to carry out the analysis of the SPC model is developed at https://gitlab.com/pjbirrell/real-time-mcmc. Code for the implementation of the strain-specific model can be found at https://github.com/anastasiachtz/COMMAND_stan/blob/master/MultiStrainStan.Rmd.

## Abbreviations

GP: General practice
HAI: Haemagglutination inhibition
HDU: High-dependence unit
ICU: Intensive care unit
ILI: Influenza-like illness
ILI+: Influenza-like illness multiplied by virological swab positivity
ISO: International organization for standardization
NHS: UK national health service
PHE: Public health England
RCGP: Royal College of general practitioners
*R*_*e*_: Effective reproduction number
RSC: Research and surveillance centre (of the RCGP)
SEIR: Susceptible-exposed-infectious-recovered (with variation in the number of ‘E’ and ‘I’s used)
SPC: Stratified primary care surveillance model
SS: Strain-specific
USISS: UK Severe Influenze surveillance system
