# Supplementary material for: Forecasting the 2017/2018 seasonal influenza epidemic in England using multiple dynamic transmission models: a case study
Source: BMC Public Health. 2020 Apr 15;20:486. doi: 10.1186/s12889-020-8455-9 (PMC7158152; doi:10.1186/s12889-020-8455-9)
Supplement: Supplementary file 1 — Additional file 1 Web extra material. Appendix to this manuscript containing technical details of each of the models discussed here, some additional results and a sensitivity study on the impact of serological information. [file 12889_2020_8455_MOESM1_ESM.pdf]

A **Appendix**: Data Availability

Over the course of the 2017-8 influenza season, the suite of data made available for modelling use evolved slightly, becoming more granular as the season progressed and modelling requirements became more apparent. By the beginning of 2018, most of the routine data streams were providing weekly data updates, but as can be seen from Figure S1, the regional-specific ILI data and swabbing data were identified later (from 2018w2).

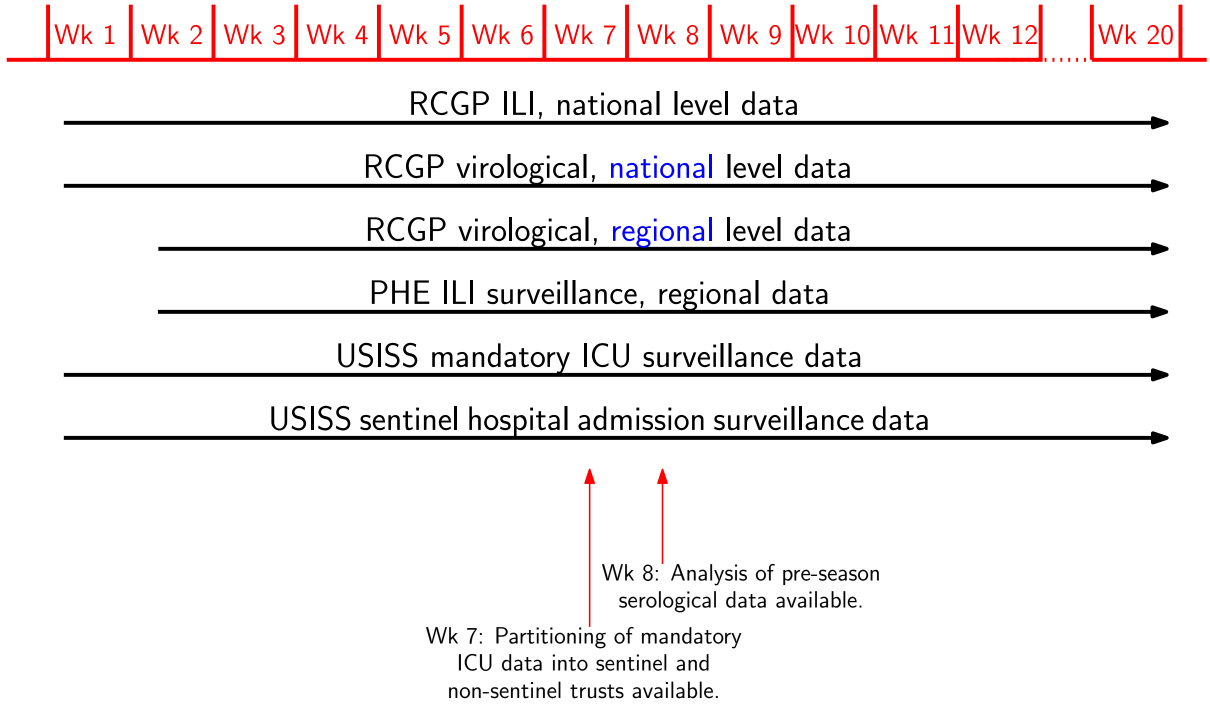

Figure S1 Timeline showing the timing of the availability of the various data items over the course of the 2018 seasonal influenza modelling period.

During the course of the influenza season, the analysis of some pre-season serological samples was conducted and finalised during week 8 of 2018, giving more precise information on the levels of immunity existing within the population prior to the influenza season. Furthermore, from week 7 onwards, it was possible to identify which of the ICU admissions in the USISS mandatory dataset came from sentinel hospital trusts. This improved the ability to estimate the ratio of ICU admissions to hospitalisations at all levels of care. The results presented in the main manuscript focus on the analyses that were carried out in “real time” during the epidemic season and, in many cases, these new information sources have been used to generate alternative analyses investigating sensitivity to the initial assumptions. For example, all models had assumed initially that the proportion of people immune to infection was equal to or around 10%. The serological data subsequently suggested that the true proportion was somewhat higher at about 40%. Consequently, and except where stated, the analyses in the main manuscript have continued to work with the initial assumption, and a discussion of additional results obtained using higher levels of immunity are presented in Appendix D.

B **Appendix:** Model Details

In what follows, all references to weeks will be to the ISO standard definition for week numbers, and a shorthand will be employed, where 2018w4 refers to ISO week 4 of 2018.

B.1 Stratified Primary Care (SPC) model

The stratified primary care model has been presented elsewhere as the parallel-region model of Birrell et al (2016). In summary, a deterministic model is used to approximate influenza transmission resulting in a time series of daily numbers of new infections. These infections then link into a disease reporting model which accounts for the proportion of the infections that are observed through consultation with a general practitioner (GP) for influenza-like illness (ILI) as well as the time period from infection to the reporting of the GP consultation. Data on GP ILI consultations are not disease specific and therefore are 'contaminated' with consultations for other infections. These non-influenza related consultations are referred to as background ILI cases. Virological (swab) positivity data is therefore required to understand the fraction of the daily ILI consultations that are attributable to influenza.

B.1.1 Transmission Dynamics

The dynamics are as detailed in Birrell et al (2016) with the exception that we only work here with a single age-group (due to insufficient information on age-group prior immunity). The model is stratified into five regions: Greater London, North (combining NHS regions North West, North East and Yorkshire and Humberside), Midlands and the East (combining the NHS regions Midlands and East of England), South East and South West. For a map of the regions see https://www.hee.nhs.uk/about/how-we-work/your-area.

In each region, a deterministic SEEIIR transmission model is implemented to track the seasonal influenza activity over T time intervals $[t_{n-1}, t_n) = [(n-1)\delta t, n\delta t), n = 1, \dots, T$. Within region r, the influenza dynamics are governed by a system of difference equations:

$$
\begin{aligned}
S_r(t_n) &= S_r(t_{n-1})(1 - \lambda_r(t_{n-1})\delta t), \\
E_{1,r}(t_n) &= E_{1,r}(t_{n-1})(1 - \sigma\delta t) + S_r(t_{n-1})\lambda_j(t_{n-1})\delta t, \\
E_{2,r}(t_n) &= E_{2,r}(t_{n-1})(1 - \sigma\delta t) + E_{1,r}(t_{n-1})\sigma\delta t, \\
I_{1,r}(t_n) &= I_{1,r}(t_{n-1})(1 - \gamma\delta t) + E_{2,r}(t_{n-1})\sigma\delta t, \\
I_{2,r}(t_n) &= I_{2,r}(t_{n-1})(1 - \gamma\delta t) + I_{1,r}(t_{n-1})\gamma\delta t,
\end{aligned}
\tag{1}
$$

where σ and γ are related to the mean durations of latent and infectious infection, d_L and d_I, via

$$
\sigma = 2/d_{\mathrm{L}}, \gamma = 2/d_{\mathrm{I}}. \tag{2}
$$

The infection hazards, $\lambda_r(t_{n-1})$, are found through the Reed-Frost formulation:

$$\lambda_r(t_n) = 1 - \left(1 - \frac{\kappa_{t_n} R_{0,r}\gamma}{2N_r}\right)^{I_{1,r}(t_n)+I_{2,r}(t_n)}.$$

Here κ_{t_n} is a school-holiday effect equal to 1 when t_n is a time outside of a school holiday period and κ during school holidays, $R_{o,r}$ is the basic reproductive number, and N_r is the population size, both in the region r. The initial conditions for the system of equations (1) are detailed in Birrell et al (2016), but it bears pointing out that the initial pool of susceptible individuals is given by $S_r(t_0) = \pi N_r$, where parameter π gives the initial fraction of the population that are susceptible to infection. Throughout, a value of $\pi = 0.9$ will be used to represent a belief that at most people will be susceptible to at least one of the circulating influenza viruses.

In each time interval $[t_{n-1}, t_n)$, a number of new infections is calculated as

$$\Delta_r(t_n) = S_r(t_{n-1})\lambda_r(t_{n-1})\delta t,$$

forming the input to the disease reporting model.

B.1.2 Disease Reporting Model

The disease reporting component of the SPC model accounts for

- the fraction of infections that lead to a GP consultation;
- the delay time from infection to the reporting of the GP consultation.
- the level of GP consultations for ILI not directly attributable to influenza infection.

It is assumed that a fraction of the infections, θ, will develop ILI symptoms. Of those who develop symptoms, it is assumed that a further fraction, p_{GP}, will consult with their GP. Where a consultation occurs, the time between infection and the reporting of the consultation is governed by a discretised gamma distribution, with parameters a and s, such that $f(k; a, s)$ gives the probability that the time from infection to the reporting of the healthcare event is $k\delta t$ (assuming that infections and disease reporting occur at the same point in each time interval). The number of GP consultations attributable to influenza infection is therefore given by

$$\mu_r^{\text{ILI+}}(t_n) = \theta p_{GP}(t_n) \sum_{k=0}^{n} f(k; a, s)\Delta_r(t_{n-k}). \quad (3)$$

In 2018w1, when we only have a single region and do not directly incorporate the virological data into the analysis, the ILI+ data are assumed to be imperfect observations of the $\mu_r^{\text{ILI+}}(t_n)$, and therefore are assumed to be distributed with (3) as their mean.

However, from 2018w2 onwards, the full GP ILI consultation data are used with virological swabbing data. As the data arrive daily, there is also a strong within-week pattern to the consultations: there is little to zero reporting of consultations at weekends, whilst many will delay their consultation until the following Monday, the busiest day of the week.

B.1.2.1 Background Consultations

The background consultations give a level of GP consultation for ILI that would exist in the absence of influenza activity. These consultations are assumed to be highly seasonal, with a peak in mid-winter. To account for this pattern, we assume a model for $B_r(w)$, the weekly rate of non-influenza ILI consultations in region r during week w, that can be expressed as:

$$\log\{B_r(w)\} = \zeta_r + \xi_r \sin\left(\frac{2\pi w}{52}\right) + \varsigma_r \cos\left(\frac{2\pi w}{52}\right);$$

the type of seasonal model used for the endemic component in the HHH model of Held, Höhle and Hofmann, 2005. All three parameters, ζ_r, ξ_r and ς_r, will be estimated, but will be done so with informative priors placed on them. These informative priors were found by taking the posteriors obtained when fitting the epidemic/endemic HHH model to GP In-Hours ILI consultation data over the period 12/1/2015 to 1/10/2017. If $W_{r,w}$ is the GP ILI consultation data in week w, then:

$$\mathbb{E}W_{r,w} = e^{\alpha_r} W_{r,w-1} + \frac{N_{r,w}}{N_r} B_r(w),$$

where $N_{r,w}$ is the catchment (observable) population in region r in week w, an average of the daily catchment populations, and α_r is the region-specific parameter of the epidemic component of the HHH model.

B.1.2.2 Day-of-the-week reporting

If we denote $d(t_n) = 1, \dots, 7$ to be the day of the week on which the nth interval falls, and, similarly, $w(t_n)$ to be the week in which the nth interval falls, then the day of the week effects upon reporting can be accounted for by introducing scalars c_d such that

$$\mu_r(t_n) = c_{d(t_n)} \left(\mu_r^{ILI+}(t_n) + \frac{B_r(w(t_n))}{7} \delta t \right) \text{ and } \prod_{d=1}^{7} c_d = 1,$$

where the product constraint ensures identifiability.

B.1.3 Likelihood

Denote

- X_{r,t_n} to be the random variable representing the observed number of ILI consultations in region r at time t_n, with observed value x_{r,t_n}.
- Y_{r,t_n} to be the random variable representing the observed number of swabs testing positive for the presence of influenza out of a sample of size K_{r,t_n} in region r at time t_n, with observed value y_{r,t_n}.

It is assumed that the ILI consultation data are distributed according to the negative binomial distribution:

$$X_{r,t_n} \sim \text{NegBin}\left(\frac{N_{r,t_n}}{N_r}\mu_r(t_n), \eta_r\right), \quad (4)$$

where the N_{r,t_n} is the size of the catchment population on day t_n, and η_r is a dispersion parameter such that $\text{var}\left(X_{r,t_n}\right) = (1 + \eta_r)\mathbb{E}X_{r,t_n}$.

Similarly, the swab positivity data are distributed

$$Y_{r,t_n} \sim \text{Bin}\left(K_{r,t_n}, \frac{\mu_r^{ILI+}(t_n)}{\mu_r^{ILI+}(t_n) + B_r\left(w(t_n)\right)\delta t/7}\right). \quad (5)$$

These distributional assumptions help us to compile a likelihood. If we denote Φ to be a vector of all the model parameters, then the likelihood is

$$L(x_r, y_r; \Phi) = \prod_{n=1}^{T} L\left(x_{r,t_n}; \Phi\right)L\left(y_{r,t_n}; \Phi\right). \quad (6)$$

For ease of presentation, equations (4)-(6) assume that the data arrive at intervals of δt. In practice, data are daily, whereas $\delta t = 1/2$ days. In which case, in (4) and (5) the $\mu_r^{ILI+}(t_n)$, $B_r(t_n)$ and $\mu_r(t_n)$ are summed to give daily totals, and the product in (6) is over days, not intervals.

B.1.4 Model Evolution

Throughout the influenza season, there were changes in the available information (see Appendix A: Data). Therefore, as with the other models, some increase in the model complexity and detail was possible over time. The table below indicates how this changed for the SPC model.

Week	Description	Date
1	ILI+ data used as the primary care data stream. Single region model combining all data.	2018-01-07
2	ILI+ replaced by PHE influenza surveillance data and RCGP swabbing data. Model is regionally stratified.	2018-01-14
8	Immunity derived from serological data. Used here for the purposes of sensitivity analysis only, using $\pi = 0.625$.	2018-02-25

B.2 Strain Specific SIR model

The model used here has an SIR structure, fitted to both the weekly ILI and virological data from RCGP. It is assumed that there are three circulating strains each acting independently.

The SIR model is specified in continuous time by the system:

$$\begin{aligned}\frac{dS_s}{dt} &= -\beta_s \frac{I_s(t)}{N} S_s(t) \\ \frac{dI_s}{dt} &= \beta_s \frac{I_s(t)}{N} S(t) - \gamma I_s(t) \\ \frac{dR_s}{dt} &= \gamma I_s(t),\end{aligned} \tag{7}$$

where $S_s(t)$ represents the number of individuals susceptible to strain s at time t. Similarly, $I_s(t)$ and $R_s(t)$ represent the numbers of infected and recovered individuals respectively at time t and for strain s. N is the total population size ($N = S_s + I_s + R_s$), β_s is the strain specific rate of transmission and γ is the daily rate of recovery. In practice, this system of equations is solved using a first-order Euler approximation with time-steps of $\delta t = 1$ day.

We denote the number of ILI attributable to influenza strain s in week w as $ILI^{+,s}(w)$, and the corresponding level of non-influenza ILI as $ILI^{-}(w)$. The total number of ILI cases in week w is then the sum of the two components i.e. $ILI(w) = ILI^{-}(w) + \sum_s ILI^{+,s}(w)$, where these components result from

$$\begin{aligned} ILI^{+,s}(w) &= \sum_{n=7(w-1)+1}^{7w} \theta_s^{+} \gamma I_s(t_n) \\ ILI^{-}(w) &= \sum_{n=7(w-1)+1}^{7w} \theta^{-}(t_n) \left(N - \sum_{s=1}^{3} I_s(t_n) \right) \end{aligned} \tag{8}$$

with θ_s^{+} the daily rate of becoming symptomatic due to influenza and θ^{-} the corresponding rate of developing ILI symptoms when not infected with influenza.

As in the SPC model, the background influenza-negative ILI rates increase during the winter (Fleming & Elliot, 2008). To account for this, θ^{-} was assumed to change over time as follows:

$$\log(\theta^{-}(t)) = \hat{\theta} - \phi \left(1 - \exp\left(-\frac{(t-\tau)^2}{2\omega^2} \right) \right), \tag{9}$$

where $\hat{\theta}$ is the maximum value of the (log) influenza negative ILI rate, ϕ is the amplitude of oscillation, τ gives the timing of the peak and ω governs the period of fluctuation. Note that the rate of influenza positive ILI is assumed to be proportional to the rate at which individuals recover from infection (see first line of Equation (8)). This introduces a delay between becoming infected and developing symptoms, visiting the GP and being diagnosed. For simplicity, we assumed that this delay is on average the same as the delay between becoming infected and recovery.

B.2.1 Likelihood

Again, assume that the observed number of ILI consultations, x_w^{ILI}, is the realisation of a random variable X_w^{ILI}, with the following distribution:

$$X_w^{ILI} \sim \mathrm{Bin}\left(\frac{N_w}{N} ILI(w), \epsilon \right),$$

where the weekly catchment population is N_w, and ϵ is the probability with which someone with ILI is diagnosed, i.e. this is a combination of the probability that the infected cases consult the GP and the GP correctly diagnoses ILI.

The virological data identify the presence of the three competing influenza strains in the ILI consultation data. In week w, these data are denoted as $y_w = \left(y_w^{+,H1}, y_w^{+,H3}, y_w^{+,B}, y_w^{-}\right)$ and they are assumed to be the realisation of a multinomial distribution:

$$Y_w \sim \text{Multi}\left(K_w, \frac{1}{ILI}\left(ILI^{+,H1}(w), ILI^{+,H3}(w), ILI^{+,B}(w), ILI^{-}(w)\right)\right),$$

where K_w represents the virological sample size in week w. The likelihood is then formed in a similar fashion to Equation (6).

B.2.2 Model evolution

Two different models have been fitted over time as new data became available. The table below describes the scenarios considered and the dates from which the analyses were carried out. Scenario 2 involves a time-dependent influenza negative ILI rate, while in Scenario 1 the influenza negative ILI rate was kept constant.

Week	Scenario	Description	Date
1	1	θ^{-} kept constant	2018-01-07
4	2	θ^{-} time dependent, according to Equation (9).	2018-01-28

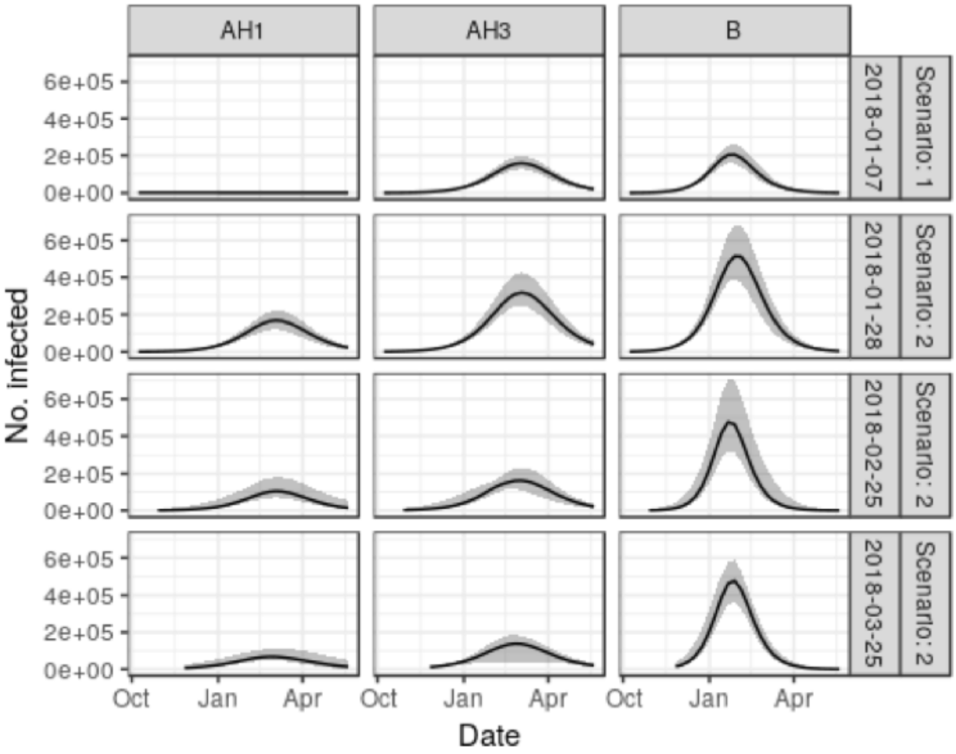

Figure S2 Estimated number of infected over time for the different strains by analysis date and type of model (see Table above).

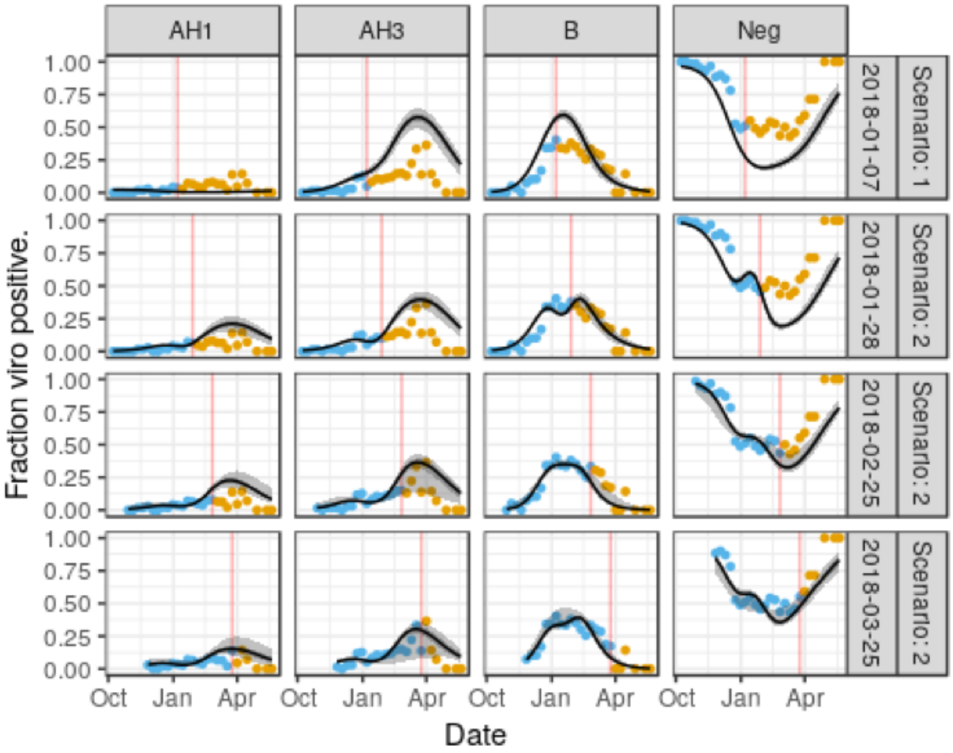

Figure S3 Model fit and data. Blue points represent the data used in the fitting, and yellow points are the data points yet to be observed at the time of analysis. The red line highlights the timing of the last data point used in the model fitting.

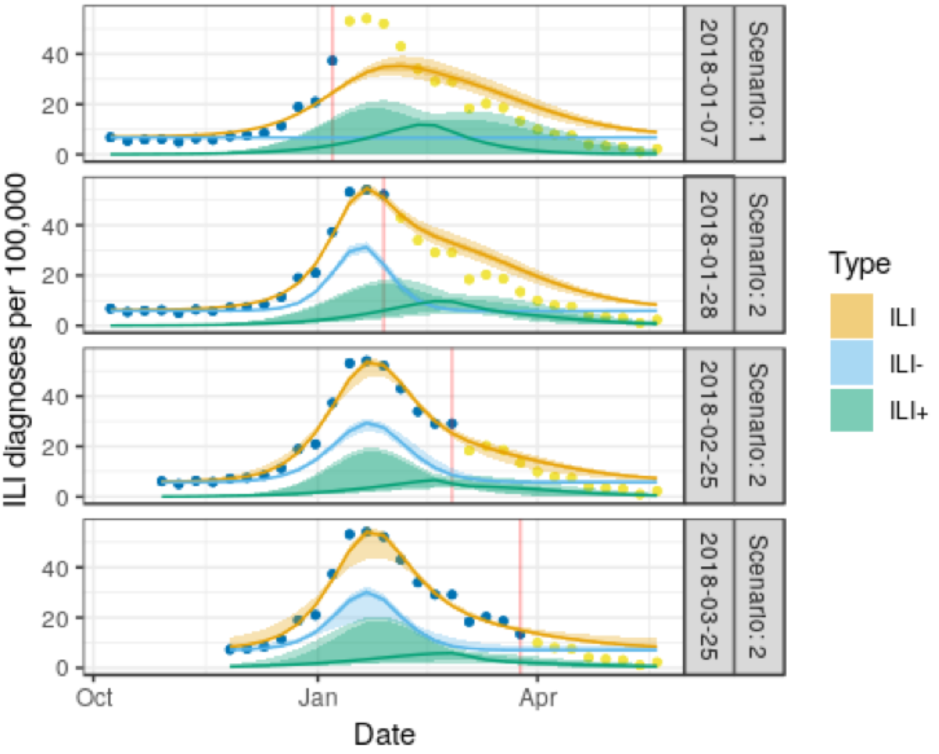

Figure S4 ILI data and model predictions. Blue points represent the data used in model fitting; yellow points are the data points yet to be observed at the time of analysis. The yellow ribbon corresponds to the total ILI rate, while the blue and green refer to the negative and positive influenza ILI, respectively. Note that for the first scenario we assume the influenza negative rate to be constant over time and the blue ribbon reflects this assumption.

B.3 ICU model

The ICU model was initially formulated to analyse data collected by USISS during the 2012/13, 2013/14 and 2014/15 influenza seasons. For a detailed description of the model we refer to Corbella et al. (2018) and we report below only the key elements and assumptions.

B.3.1 Transmission model

We assume that the transmission dynamics can be approximated by a continuous-time deterministic SEEIIR model:

$$\begin{aligned}
\frac{dS}{dt} &= -\beta(t)\frac{I_1 + I_2}{N}S \\
\frac{dE_1}{dt} &= \beta(t)\frac{I_1 + I_2}{N}S - \sigma E_1 \\
\frac{dE_2}{dt} &= \sigma E_1 - \sigma E_2 \\
\frac{dI_1}{dt} &= \sigma E_2 - \gamma I_1 \\
\frac{dI_2}{dt} &= \gamma I_1 - \gamma I_2,
\end{aligned} \tag{10}$$

where σ and γ relate to the mean latent and infectious periods respectively as in Equation (2).

The transmission rate $\beta(t)$ is assumed to change over time as follows:

$$\beta(t) = \begin{cases} \kappa\beta_0, & t \in \text{school holidays} \\ \beta_0, & \text{otherwise.} \end{cases} \tag{11}$$

The factor $\kappa \in (0,2)$ allows for the possibility that rates of infectious contact might change as a result of school holidays. Beyond this, it is assumed that contact and transmission are homogeneous over age, geographical region and influenza sub-type. At 2018w8 a number of alternative models were considered, models assuming:

- a constant transmission rate: $\beta(t) = \beta_0$
- only a Christmas holiday effect:
$$\beta(t) = \begin{cases} \kappa\beta_0, & t \in \text{Christmas holiday,} \\ \beta_0, & \text{otherwise.} \end{cases}$$
- transmission dependent on climate variables: $\beta(t)$ assumed to be a linear function of either the daily absolute humidity or of the daily temperature, denoted below by $c(t)$
$$\beta(t) = \beta_0 + \beta_1 c(t)$$

The competing models were used to analyse all the data up to 2018w8 inclusive. The Deviance Information Criterion (DIC) (Spiegelhalter et al, 2002) was then used to compare the possible models. The best-fitting model according to the DIC was the school-holiday model (Equation (11)). The other models were not considered further.

B.3.2 Reporting model

Denote by:

- $\Delta(w)$ the number of new infections during week w;
- p_{ICU} the probability of Intensive Care Unit (ICU) admission given infection;
- $f_{ICU|INF}(u)$ the probability of u weeks elapsing between infection and ICU admission;
- $d(w)$ the probability of detection given ICU admission at week w.

The number of people detected during week w, $\mu(w)$, is, similarly to Equation (3):

$$\mu(w) = d(w)p_{ICU} \sum_{v=0}^{w} \Delta(v) f_{ICU|INF}(w - v)$$

Lastly, we assume that y_w^{ICU} , the number of ICU admissions in week w is the realisation of a random variable Y_w^{ICU} which has a Negative Binomial distribution centred on $\mu(w)$ with over-dispersion parameter η^{ICU}:

$$Y_w^{ICU} \sim \text{NegBin}(\mu(w), \eta^{ICU}).$$

B.3.3 Inference

We assume the latent and infectious periods have fixed means $d_L = 2$ and $d_I = 3$, respectively. Moreover, we assume that the weekly probability of detection $d(w)$ is known and equal to the known fraction of the total population within the catchment of reporting hospitals that reported data over the total monitored population, i.e.,

$$d_w = \frac{N_w}{N}$$

where N_w is the total population within the catchment of reporting hospitals in week *w* and *N* is the full population of England.

Like the other modelling approaches, inference is carried out within the Bayesian paradigm: prior distributions for model parameters are assumed to be uniform within some plausible limits, as described in Corbella et al. (2018), with the exception of the initial susceptibility, π, and p_{ICU} which are distributed:

$$\pi \sim \text{Beta}(5, 45)$$
$$p_{ICU} \sim \text{LogNormal}(\log(\mu) = \log(0.00012), \sigma = 0.51).$$

Approximate posterior distributions are found using the Metropolis Hastings algorithm described in the appendix of Corbella et al. (2018).

B.4 Synthesis model

The synthesis model was proposed as a parsimonious attempt to utilise ILI, virological (combined as ILI consultations × swab positivity, to give a dataset labelled ILI$^+$), hospitalisation and ICU data to jointly infer and to predict the transmission dynamics of 2017/18 seasonal influenza outbreak. The schematic representation of the model is shown in the following figure:

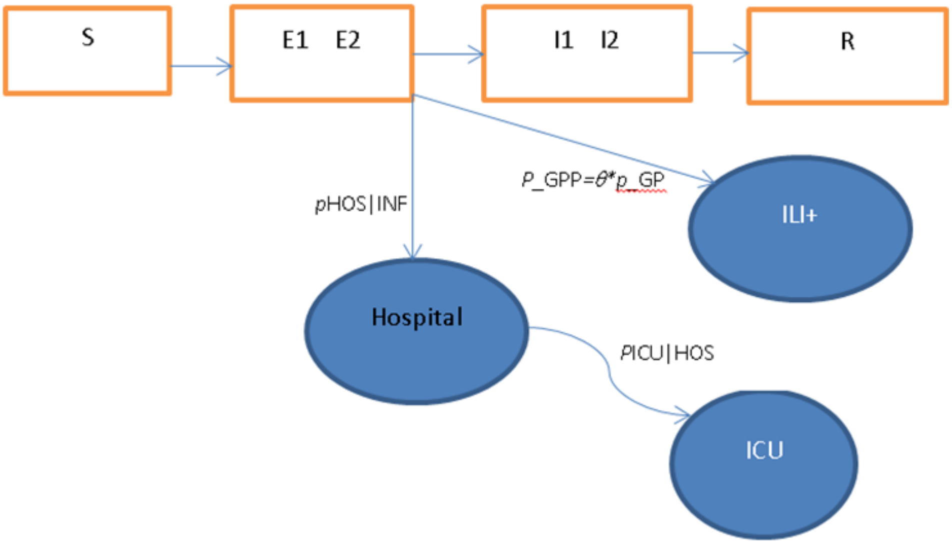

Figure S5 Diagram of synthesis model in which a SEEIIR transmission model dynamics are informed by observations of ILI+, Hospital and ICU admission data (the shaded circle boxes).

B.4.1 Transmission Dynamics

Again, SEEIIR transmission dynamics are assumed:

$$\begin{aligned}
\frac{dS}{dt} &= -\beta\frac{I_1(t)+I_2(t)}{N}S(t) \\
\frac{dE_1}{dt} &= \beta\frac{I_1(t)+I_2(t)}{N}S(t) - \sigma E_1(t) \\
\frac{dE_2}{dt} &= \sigma\big(E_1(t)-E_2(t)\big) \\
\frac{dI_1}{dt} &= \sigma E_2(t) - \gamma I_1(t) \\
\frac{dI_2}{dt} &= \sigma\big(I_1(t)-I_2(t)\big).
\end{aligned} \tag{12}$$

As with the SPC model, the reproductive number is linked to an initial rate of exponential growth, with rate ψ,

$$R_0 = {}^{2\beta}/_{\gamma} = \frac{2\psi}{\gamma}\frac{\left({}^{\psi}/_{\sigma}+1\right)^2}{1-\frac{1}{\left({}^{\psi}/_{\gamma}+1\right)^2}}.$$

Here, the mean infectious period $\left({}^{2}/_{\gamma}\right)$ and the mean latent period $\left({}^{2}/_{\sigma}\right)$ are both held fixed at 3.47 and 2.0 days respectively (Birrell et al 2011).

B.4.2 Disease and reporting processes

The disease and reporting component of the model is the component coloured blue in Figure S5. The following three parameters describe the proportion of infections that appear in the respective datasets:

- p_{GPP}: the proportion of infections that will lead to an influenza positive GP consultation for ILI, the product of the proportion of symptomatic infections (θ) and the proportion that consult with a GP consultation given ILI (p_{GP}) (cf. Equation 3).
- p_H: the proportion of infections that will lead to a hospital admission for ILI.
- $p_{I|H}$: the proportion of hospitalisations for ILI that lead to an ICU admission.

The proportion infections leading to an ICU admission, p_I is given by

$$p_I = p_{I|H} \times p_H,$$

so that there is an implicit assumption that all ICU admissions are initially recorded as a hospitalisation.

In week w, the value of the ILI+ data (an estimate of the number of influenza-positive GP ILI consultations) had expected value

$$\mu^{\mathrm{ILI}}(w) = p_{GPP}\sum_{u=0}^{w} f_{ILI}(w-u; a^{ILI}, s^{ILI})\Delta_I(u).$$

In a slight variation from the ICU and SPC models, $\Delta_I(u)$ represents the weekly number of newly infectious (not infected) cases in week u. Correspondingly, f_{ILI} gives the appropriate quantiles of a gamma delay distribution, with $f_{ILI}(w-u;\cdot,\cdot)$ describing the probability that the reporting of an ILI consultation occurs $w-u$ weeks after an individual becomes infectious. The parameters governing this gamma distribution are those governing the distribution of the delay from symptom onset to GP consultations and are taken from Birrell et al (2011) to be $a^{ILI} = 3.06$ and $s^{ILI} = 10.22$ (when multiplied to a weekly, rather than daily, scale). In this study we simple assume that the incubation is equal to the latent period.

The weekly number of new hospital admissions at week w was similarly generated as

$$\mu^{\mathrm{H}}(w) = p_H\sum_{u=0}^{w} f_H(w-u; a^H, s^H)\Delta_I(u).$$

Here f_H is parameterised by $a^H = 0.708$, $s^H = 1.813$ from the assumed distribution of delay times from infectiousness onset to hospital admission.

The weekly number of new ICUs at week w was generated as

$$\mu^{\mathrm{ICU}}(w) = p_{I|H} \sum_{u=0}^{w} f_{I|H}(w-u; a^{ICU}, s^{ICU}) \mu^H(u).$$

Here $f_{I|H}$ is parameterised by $a^{ICU} = 0.425$, $s^{ICU} = 2.163$ from the assumed distribution of delay times from hospital admission to ICU admission. The parameters used for the above two delay distributions have been estimated on the basis of a simple analysis of USISS data from the 2012/13 and 2013/14 influenza seasons (not shown).

B.4.3 Inference

To reflect the over-dispersion in the weekly ILI+ numbers, the negative binomial likelihood is still assumed even though there is no guarantee that the ILI+ data, Y_w^{ILI+}, will be integer-valued. The observed number, y_w^{ILI+}, within a catchment population in week w of N_w has probability density

$$l(y_w^{ILI+}|\Phi) = \frac{\Gamma\left(y_w^{ILI+} + r(w)\right)}{\Gamma\left(r(w)\right)\Gamma(1 + y_w^{ILI+})} \left(\frac{1}{\eta^{ILI+}}\right)^{r(w)} \left(1 - \frac{1}{\eta^{ILI+}}\right)^{y_w^{ILI+}}$$

where

$$r(w) = \frac{N_w}{N} \frac{\mu^{\mathrm{ILI+}}(w)}{\eta^{ILI+} - 1}.$$

Here η^{ILI} is the dispersion parameter. Similarly, and more straightforwardly, the hospitalisation and ICU data, y_w^H and y_w^{ICU}, also follow negative binomial distributions with dispersion parameters: η^H, η^{ICU} respectively.

Assuming independence across the datasets and independent observation errors over time (conditional upon model parameters), the total likelihood given model parameter Φ is

$$L\left(y_{1:W}^{ILI+}, y_{1:W}^{H}, y_{1:W}^{ICU}; \Phi\right) = \prod_{w=1}^{W} L(y_w^{ILI+}; \Phi) L(y_w^H; \Phi) L(y_w^{ICU}; \Phi).$$

Uninformative prior distributions are chosen for all model parameters (see Table S1). Again, posterior distributions are obtained via Markov Chain Monte Carlo simulations (MCMC). From these samples, we can obtain means and 95% credible intervals for model parameters.

In the followings we show the model predictions using the model calibrated at four different weeks: 2018w1, 2018w4, 2018w8 and 2018w12. These results were obtained from 5000 MCMC samples.

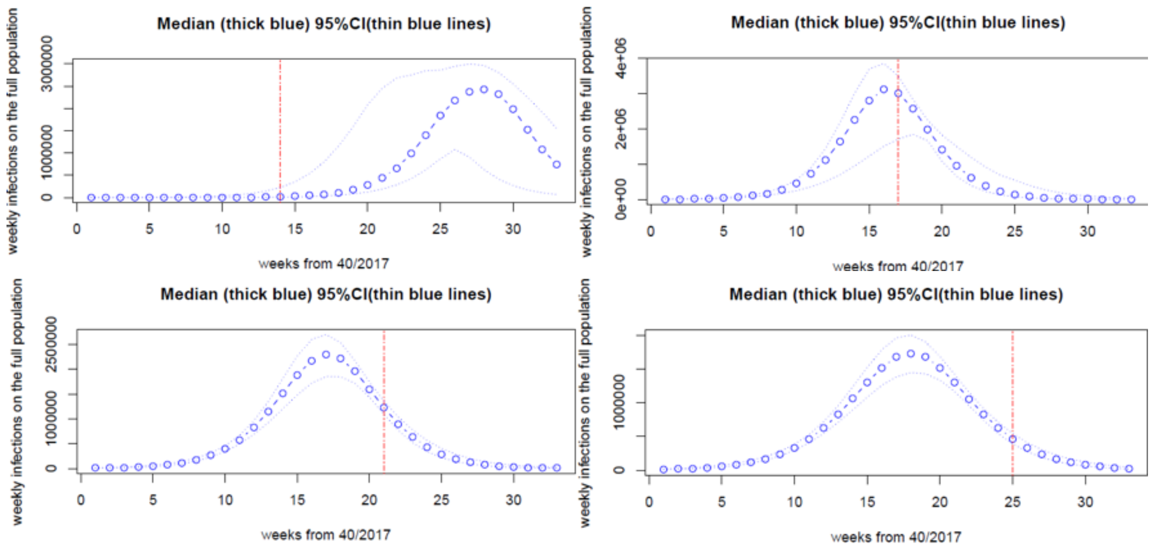

Figure S6 Synthesis model predictions of weekly number of infections from 2018w1, 2018w4, 2018w8, and 2018w12. The thick blue line joining the circles represent posterior medians with the thinner lines giving the 95% credible interval. The red vertical line labels the latest week included in the data used to calibrate the model.

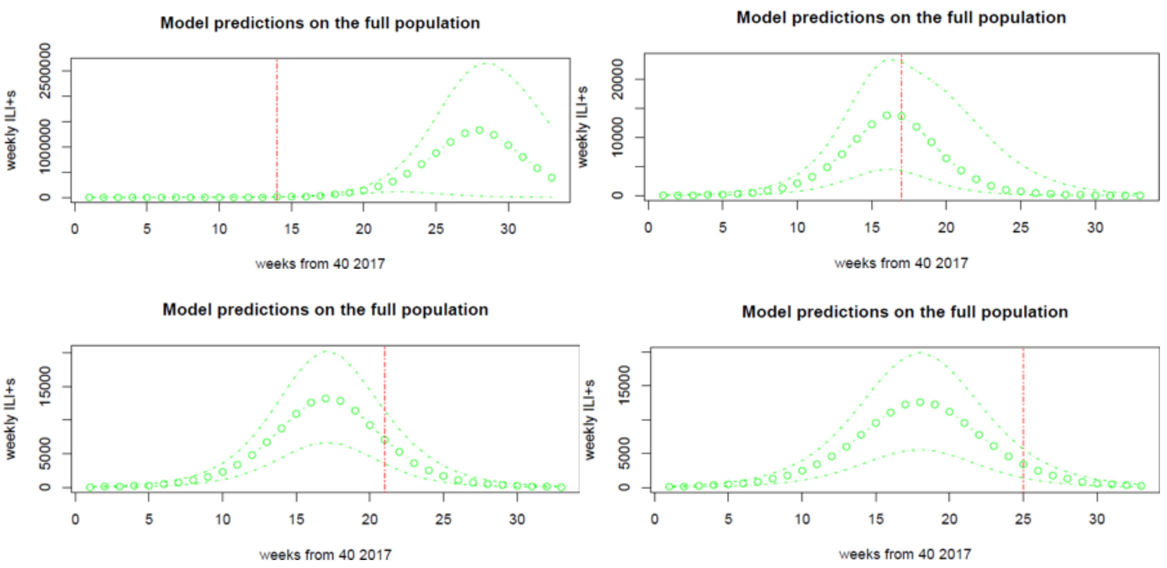

Figure S7 Synthesis model predictions of weekly number of ILI+ from 2018w1, 2018w4, 2018w8 and 2018w12. The thick green line and circles indicate the posterior medians. The thin green dotted lines give the 95% credible intervals. The red vertical line labels the latest week included in the data used to calibrate the model.

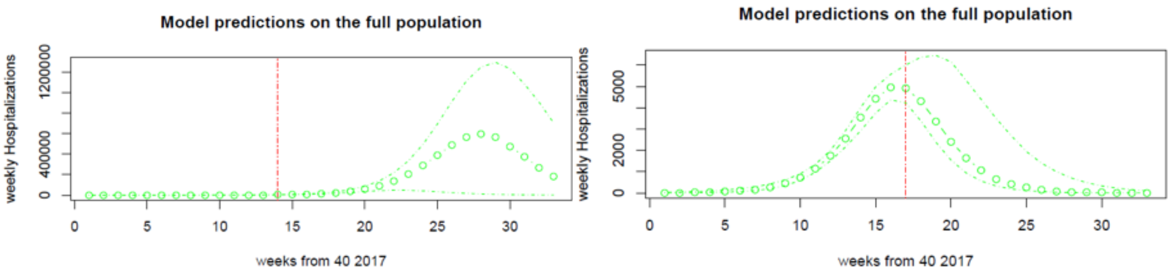

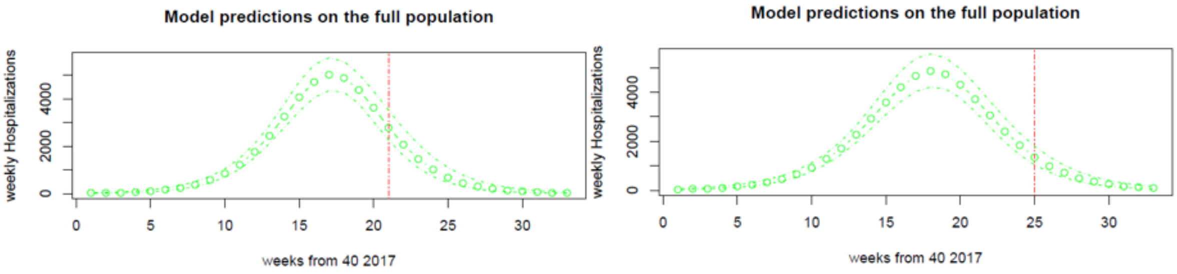

Figure S8 Synthesis model estimates of the weekly number of hospitalisation admissions from 2018w1, 2018w4, 2018w8 and 2018w12. The thick green lines and circles represent the posterior means and the dashed lines the 95% credible intervals.

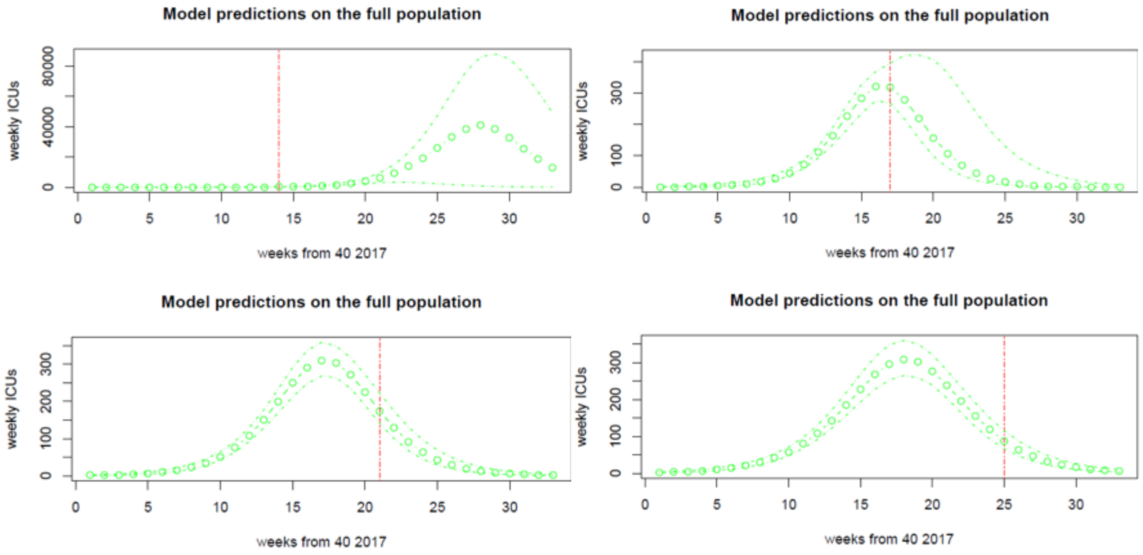

Figure S9 Synthesis model estimates of weekly number of ICU admissions from 2018w1, 2018w4, 2018w8, and 2018w12. The thick green lines and circles represent posterior medians and the thin dashed lines give 95% credible intervals.

B.5 Prior Distributions and Model Assumptions

Table S1 gives a comparison of the assumed parameter values and, where the parameter is to be estimated, prior distributions for each of the four modelling approaches.

Table S1 Assumptions and structure of four models. Some model parameters were assumed the values from previous studies and others were estimated via the collected data until the date of exercise. [a]Delay distribution for GP ILI data in SPC and synthesis models from Birrell et al (2011); [b]Delay distribution for USISS data from seasons 2011/12, 2012/13; [c]Prior for SS model, taken from Biggerstaff et al 2014; [d]Prior for SS model, taken from Carrat et al 2008; [e]The initial susceptibility rate is derived from serological data; [f]Prior for SS model, taken from van Noort et al 2015.

Name	SPC model	Strain-Specific model	ICU model	SYNTHESIS model
Compartmental structure	SEEIIR	SIR	SEEIIR	SEEIIR
Continuous/discrete	Discrete (dt=0.5day)	Discrete (dt=1 day)	Continuous	Discrete (dt=0.25 day)
Heterogeneity in contact/transmission	Region/calendar time	strain	Calendar time	–
Observational noise	Negative Binomial for ILI, binomial for virological data.	Binomial for ILI data<br><br>Multinomial for virological data	Negative Binomial	Negative Binomial for ILI+, Hospitalizations, and ICU data
Data	Daily GP in hours ILI consultations<br><br>Daily RCGP virological swabbing data	Weekly RCGP GP ILI consultations<br><br>Weekly RCGP virological data	Weekly USISS ICU admissions	Weekly GP ILI, RCGP Positivity, Hospitalizations, and ICU admissions
Background ILI	Yes	Yes	n/a	n/a
Fixed parameters and quantities	Initial proportion susceptible (π= 0.9)<br><br>Mean latent period 2days,	n/a	Mean latent period 2 days,<br><br>Infectious period 3.5 days.	Initial proportion susceptible (π= 0.9)<br><br>Infectious period 3.5 days

	Proportion of symptomatic infections 0.15<br><br>Delay from infection to the reporting of a GP ILI consultation (in days) $\sim\Gamma(1.37, 0.336)$		Time to symptom onset $\sim\Gamma(0.678, 0.417)$ (from Tom et al. 2014).<br><br>Time to ICU admission from symptom onset $\sim \log N(\log(0.607), 1.022)$	Mean latent period 2 days<br><br>Delay from symptom onset to GP $\sim\Gamma(3.06, 1.46)$[a]<br><br>Delay from symptom onset to HOS $\sim\Gamma(0.708, 0.259)$[b]<br><br>Delay from Hospitalization to ICU $\sim\Gamma(0.425, 0.309)$[b]
Priors for parameters to be estimated	Exponential growth rate ~U(0,0.25)<br><br>Average infectious period ~U[2,4] days.<br><br>Contact matrix parameters m_i ~U(0,1)<br><br>Propensity to consult with a GP p_{GP} ~ U(0, 0.4)<br><br>ν=log(pλ) ~ U(-20,-10)<br><br>Day of week of effects in GP consultation and background GP consultation rates are 6 and 3 dimensional parameters with multivariate normal priors.<br><br>Region-specific dispersion parameters, $\eta_r \sim \Gamma(0.01, 0.01)$.	R_{eff} ~ N(1.28,0.133)[c]<br><br>Infectious period ~ N(4.8,0.245)[d]<br><br>Symptomatic rates (θ_s^+): $\theta_{H1}^+ \sim \beta(18.21, 30.61)$, $\theta_{H3}^+ \sim \beta(36.42, 52.98)$, $\theta_B^+ \sim \beta(4.55, 50.90)$[d]<br><br>Susceptibility (π) ~ N(0.8,0.3)[e]<br><br>Visitation rate(ε)~ Beta(35.644,69.314)[f]	Initial susceptibility π ~Beta(45,5)<br><br>$p_{ICU\|INF}$ ~LogN(log(0.00012),.51)<br><br>All the other priors are assumed uniform in the range used in Corbella et al 2018.	Exponential growth rate ~U(0.1,1.4)<br><br>Initial seed U(1,5000)<br><br>P_GPP ~ U(.0001,1.0)<br><br>$p_{HOS\|INF}$ ~U(0.0001,0.75)<br><br>$p_{ICU\|HOS}$ ~U(0.0001,0.1)<br><br>η_{ILI+}, η_{HOS} η_{ICU} ~ U(1.01,500)

C **Appendix**: Results tables

Table S2 Posterior median (95% credible interval) for R0, Re, and ILI+, HOS and ICU rates among 100,000 infections. Abbreviations: GL = Greater London; Nth = North; SE = South East; SW = South West; Mids = Midlands and East. Across all models, the initial proportion susceptible is fixed at 0.9 unless explicitly stated in the text. Note that the SPC model has a day-specific proportion of cases that report a GP consultation, as this quantity absorbs the day-of-the-week effect in reporting rates.

**Week of Analysis**	**Model**	R_0	R_e	**ILI+ rate**	**HOS rate**	**ICU rate**
2018w1	SPC	1.54 [1.45, 1.64]		3826 [1032, 5873]	–	-
	SS	1.74 [1.28, 2.52] (AH1)<br>5.02 [4.66, 5.47] (AH3)<br>3.97 [3.72, 4.25] (B)	1.19 [1.16, 1.21] (AH1)<br>1.56 [1.54, 1.59] (AH3)<br>1.72 [1.69, 1.75] (B)	1001 [835, 1183]	–	
	ICU	1.44 [1.33,1.63]	1.30 [1.24,1.36]	-	–	12.55 [5.9,30.9]
	Synthesis	1.49 [1.44,1.52]	1.34[1.30,1.37]	52562 [4166,97850]	23660 [1868,48635]	1614(128,33646]
2018w4	SPC	1.62[1.53,1.73] (GL)<br>1.83[1.71,1.99] (North)<br>1.78[1.66,1.95] (SE)<br>1.79[1.67,1.95] (SW)<br>1.69[1.58,1.82] (Mids)		630 [558,715] (Mon)<br>471 [415,535] (Tue)<br>468 [413,531] (Wed)<br>419 [370,476] (Thu)<br>462 [408,523] (Fri)<br>16 [13,21] (Sat)<br>4 [3,6] (Sun)	–	
	SS	2.69 [1.86, 3.64] (AH1)<br>2.65 [1.92, 3.36] (AH3)<br>1.80 [1.68, 2.04] (B)	1.55 [1.53, 1.58] (AH1)<br>1.55 [1.53, 1.58] (AH3)<br>1.68 [1.65, 1.71] (B)	401 [315, 466]	–	
	ICU	1.46 [1.36,1.63]	1.32 [1.27,1.37)	-	–	12.1 [8.9,18.2]
	Synthesis	1.53[1.41,1.59]	1.38 [1.27,1.43]	445 [334,952]	163 [129,335]	10.5 [8.2,22.6]
2018w8	SPC	1.61[1.49,1.67] (GL)<br>1.85[1.66,1.95] (Nth)		605 [549,712] (Mon)<br>441 [399,519] (Tue)	–	

		1.78[1.61,1.87] (SE)<br>1.74[1.58,1.84] (SW)<br>1.65[1.52,1.73] (Mids)		433 [392,508] (Wed)<br>396 [358,464] (Thu)<br>433 [392,509] (Fri)<br>17 [13,21] (Sat)<br>6 [4,8] (Sun)		
	SS	2.93 [2.13, 3.61] (AH1)<br>4.81 [4.44, 5.23] (AH3)<br>4.19 [3.32, 4.56] (B)	1.55 [1.45, 1.59] (AH1)<br>1.54 [1.46, 1.59] (AH3)<br>1.84 [1.72, 1.87] (B)	478 [395, 546]	–	
	ICU	1.46 [1.36,1.63]	1.32 [1.29,1.35]	-	–	13.0 [11.4,15.5]
	Synthesis	1.46 [1.41,1.49]	1.31[1.27,1.34]	580 [477,736]	221 [188,267]	13.7 [11.6,16.8]
2018w12	SPC	1.53[1.43,1.57] (GL)<br>1.66[1.52,1.73] (Nth)<br>1.65[1.52,1.72] (SE)<br>1.61[1.49,1.69] (SW)<br>1.57[1.46,1.62] (Mids)		763 [701,912] (Mon)<br>558 [512,668] (Tue)<br>539 [494,644] (Wed)<br>493 [452,590] (Thu)<br>533 [491,638] (Fri)<br>22 [18,28] (Sat)<br>8 [6,11] (Sun)	–	
	SS	2.77 [2.08, 3.82] (AH1)<br>4.76 [2.87, 5.30] (AH3)<br>4.01 [2.96, 4.36] (B)	1.48 [1.38, 1.59] (AH1)<br>1.51 [1.28, 1.57] (AH3)<br>1.79 [1.69, 1.85] (B)	505 [415, 660]	–	
	ICU	1.38 [1.28,1.55]	1.25 [1.21,1.28]	-	–	19.1 [16.0,24.0]
	Synthesis	1.40 [1.37,1.42]	1.26[1.23,1.28]	723 [600,902)	282 [244,333)	18.0 [15.3,21.3]

Table S3 Estimated epidemic attack rates (cumulative incidence) as a proportion of the total population size over the period 2017w40-2018w20.

Week of Analysis	Total Attack rate			
	SPC model	SS model	ICU model	Synthesis model
2018w1	0.438 [0.374, 0.495] (Eng)	0.0023 [0.0014, 0.0047] (AH1)<br>0.13 [0.12, 0.14] (AH3)<br>0.23 [0.22, 0.25] (B)	0.370 [0.299, 0.432]	0.375 [0.115, 0.736]
2018w2	0.526 [0.464, 0.578] (GL)<br>0.618 [0.555, 0.664] (North)<br>0.611 [0.544, 0.663] (SE)<br>0.605 [0.539, 0.654] (SW)<br>0.572 [0.506, 0.624] (Mids)<br>0.587 [0.524, 0.635] (Overall)	0.0034 [0.002, 0.0056] (AH1)<br>0.14 [0.12, 0.16] (AH3)<br>0.24 [0.22, 0.26] (B)	0.370 [0.305, 0.428]	0.333 [0.111, 0.647]
2018w3	0.505 [0.454, 0.564] (GL)<br>0.600 [0.549, 0.659] (North)<br>0.604 [0.548, 0.664] (SE)<br>0.589 [0.535, 0.650] (SW)<br>0.543 [0.489, 0.605] (Overall)	0.21 [0.0075, 0.31] (AH1)<br>0.15 [0.13, 0.16] (AH3)<br>0.24 [0.22, 0.26] (B)	0.371 [0.310, 0.427]	0.213 [0.022,0.814]
2018w4	0.494 [0.440, 0.552] (GL)<br>0.595 [0.540, 0.651] (North)<br>0.575 [0.517, 0.637] (SE)<br>0.579 [0.521, 0.639] (SW)<br>0.531 [0.474, 0.590] (Mids)<br>0.555 [0.501, 0.611] (Overall)	0.22 [0.16, 0.35] (AH1)<br>0.23 [0.19, 0.32] (AH3)<br>0.47 [0.40, 0.51] (B)	0.376 [0.318, 0.436]	0.440 [0.249, 0.596]
2018w5	0.503 [0.446, 0.559] (GL)<br>0.591 [0.533, 0.646] (North)<br>0.571 [0.508, 0.631] (SE)<br>0.568 [0.504, 0.631] (SW)<br>0.533 [0.472, 0.591] (Mids)<br>0.554 [0.497, 0.608] (Overall)	0.23 [0.18, 0.32] (AH1)<br>0.23 [0.17, 0.32] (AH3)<br>0.48 [0.42, 0.53] (B)	0.374 [0.315, 0.428]	0.455 [0.368, 0.536]
2018w6	0.502 [0.437, 0.548] (GL)<br>0.594 [0.525, 0.642] (North)<br>0.568 [0.497, 0.618] (SE)<br>0.557 [0.486, 0.609] (SW)<br>0.531 [0.462, 0.580] (Mids)<br>0.553 [0.486, 0.597] (Overall)	0.24 [0.17, 0.32] (AH1)<br>0.25 [0.18, 0.35] (AH3)<br>0.49 [0.41, 0.53] (B)	0.370 [0.321, 0.422]	0.444 [0.375, 0.511]
2018w8	0.488 [0.413, 0.523] (GL)<br>0.601 [0.518, 0.638] (North)<br>0.573 [0.489, 0.612] (SE)<br>0.555 [0.473, 0.599] (SW)<br>0.515 [0.436, 0.552] (Mids)<br>0.549 [0.469, 0.580] (Overall)	0.19 [0.16, 0.28] (AH1)<br>0.12 [0.10, 0.14] (AH3)<br>0.26 [0.24, 0.31] (B)	0.387 [0.337, 0.425]	0.387 [0.319, 0.456]
2018w12	0.440 [0.366, 0.467] (GL)<br>0.518 [0.435,0.554] (North)<br>0.513 [0.430,0.546] (SE)<br>0.492 [0.410, 0.531] (SW)<br>0.468 [0.390, 0.498] (Mids)<br>0.489 [0.409, 0.512] (Overall)	0.16 [0.10, 0.25] (AH1)<br>0.11 [0.041, 0.13] (AH3)<br>0.25 [0.23, 0.3] (B)	0.322 [0.274, 0.365]	0.342 [0.291, 0.394]

Table S4 Estimated peak week and magnitude in the number of weekly infections, by week of estimation. Peak magnitude is measured as the weekly number of infections per 100 people.

Week of analysis	SPC model		SS model		ICU model		Synthesis model	
	Peak week	Peak size	Peak week	Peak size	Peak week	Peak size	Peak week	Peak size
2018w1	21 [18, 22]	5.8 [4.7, 7.1]	1 [1, 21] (AH1)<br>22 [21, 22] (AH3)<br>17 [16, 17] (B)<br>17 [16, 18] (Overall)	7.9e-03 [5.5e-03, 1.6e-02] (AH1)<br>1.1 [0.94, 1.2] (AH3)<br>2.6 [2.4, 2.9] (B)<br>3.2 [3.0, 3.5] (Overall)	15 [14, 20]	4.3 [3.1, 7.7]	28 [22, 30]	4.8 [4.0, 5.6]
2018w2	15 [15, 15]	11.7 [9.1, 12.9]	1 [1, 22] (AH1)<br>22 [20, 22] (AH3)<br>17 [17, 18] (B)<br>18 [18, 19] (Overall)	1.1e-02 [6.8e-03, 1.8e-02] (AH1)<br>1.2 [1.0, 1.4] (AH3)<br>2.5 [2.3, 2.8] (B)<br>3.4 [3.1, 3.8] (Overall)	14 [14, 20]	4.6 [3.1, 7.6]	29 [23, 31]	4.2 [3.5, 4.8]
2018w3	15 [15, 15]	11.4 [10.3, 12.5]	22 [14, 22] (AH1)<br>21 [20, 22] (AH3)<br>18 [17, 18] (B)<br>19 [18, 20] (Overall)	1.7 [2.3e-02, 2.6] (AH1)<br>1.3 [1.1, 1.5] (AH3)<br>2.5 [2.3, 2.8] (B)<br>4.8 [3.3, 5.8] (Overall)	14 [14, 20]	4.3 [3.2, 6.3]	30 [17, 33]	3.0 [2.3, 5.5]
2018w4	15 [15, 15]	10.3 [8.7, 12.1]	22 [21, 22] (AH1)<br>22 [21, 22] (AH3)<br>17 [17, 18] (B)<br>19 [18, 19] (Overall)	1.8 [1.3, 2.9] (AH1)<br>1.9 [1.5, 2.6] (AH3)<br>5.0 [4.1, 5.5] (B)<br>7.5 [6.6, 8.8] (Overall)	14 [14, 20]	4.6 [3.3, 6.3]	16 [16, 19]	5.6 [3.4, 7.0]
2018w5	15 [15, 15]	10.0 [8.7, 11.8]	22 [21, 22] (AH1)<br>22 [21, 22] (AH3)<br>17 [17, 18] (B)<br>18 [18, 19] (Overall)	1.8 [1.4, 2.6] (AH1)<br>1.9 [1.3, 2.6] (AH3)<br>5.1 [4.4, 5.8] (B)<br>7.7 [6.3, 8.4] (Overall)	14 [14, 20]	4.4 [3.3, 5.8]	16 [16, 17]	6.1 [4.8, 7.1]
2018w6	15 [15, 15]	9.6 [8.5, 10.5]	22 [21, 22] (AH1)<br>22 [20, 22] (AH3)<br>17 [16, 17] (B)<br>17 [17, 18] (Overall)	2.0 [1.4, 2.8] (AH1)<br>2.1 [1.5, 3.0] (AH3)<br>5.7 [4.8, 6.4] (B)<br>8.0 [6.9, 9.4] (Overall)	15 [14, 20]	4.0 [3.3, 5.2]	16 [16, 17]	5.7 [4.7, 6.5]
2018w8	15 [15,16]	9.1 [7.8, 9.8]	22 [21, 22] (AH1)<br>22 [18, 22] (AH3)<br>16 [16, 17] (B)<br>17 [17, 17] (Overall)	1.6 [1.1, 2.4] (AH1)<br>0.93 [0.70, 1.2] (AH3)<br>3.6 [3.0, 3.9] (B)<br>5.0 [4.5, 5.5] (Overall)	15 [14, 16]	4.4 [3.6, 5.1]	17 [17, 18]	4.2 [3.4, 4.9]
2018w12	16 [16, 16]	6.8 [5.7, 7.6]	22 [20, 22] (AH1)<br>21 [20, 22] (AH3)<br>17 [16, 17] (B)<br>17 [17, 18] (Overall)	1.1 [0.59, 2.2] (AH1)<br>0.82 [0.18, 1.1] (AH3)<br>3.3 [2.9, 3.8] (B)<br>4.7 [3.9, 5.7] (Overall)	20 [14, 20]	3.0 [2.4, 3.6]	18 [18, 18]	3.1 [2.6, 3.6]

D **Appendix:** Serological Information and Sensitivity to the Initial Susceptibility, π

The main manuscript discusses the availability of pre-season (i.e. based on samples taken prior to week 40, 2017) serological data. These data are informative as they are indicative of the levels of pre-existing immunity in the population prior to the seasonal epidemic. In practice, this tells us the number of people in the susceptible states at time-0 ($S(t_0)$ in equations 1, 7, 10 and 12). The analyses of the pre-season serological data were complete by week 8 of 2018, towards the end of the modelling period under scrutiny in this manuscript. The modelling efforts included in the main manuscript were as completed in real-time, using the best available data at analysis time. The information that came from the analysis of the serological samples was therefore not quite timely enough to be properly understood within the required timeframe and therefore we study its impact here as a sensitivity analysis.

An initial assumption had been made that 0.9 was a suitable figure to describe the proportion of the population who were susceptible to infection with any of the circulating influenza strains. As can be seen from Table S1 this was the value for the model parameter π that has been used largely throughout. However, the analysis of the serological data revealed that 37.5% of samples returned titer values indicative of the presence of antibodies to all three circulating strains. This suggests that, for the single-strain models, a value of π=0.625 for the proportion of individuals susceptible to infection. This may still be an over-estimation of the proportion susceptible to infection, as the serology samples were taken in the summer of 2017, prior to the vaccination of ~14 million people in autumn 2018. If the vaccine is effective, this is likely to have a profound impact on population immunity if the vaccine is effective.

Different models handled initial susceptibility differently. Again, from Table S1 it can be seen that the SPC and Synthesis models use a fixed value, whereas the ICU models place a prior probability distribution on this value. This parameter cannot be directly identified from data, and therefore strong prior information is required, and this is reflected in the prior choice of the ICU model. For example, the ICU model uses a prior $\pi \sim \mathrm{Beta}(45,5)$, which has a mean of 0.9 and a small standard deviation of 0.04. In the sensitivity analysis, this is replaced by $\pi \sim \mathrm{Beta}(30,20)$ which has a mean of 0.6 and a standard deviation of 0.07, comfortably covering the 0.625 value used in the SPC and Synthesis model's sensitivity analysis.

The lowering of the initial susceptibility has the following impacts:

1. Leads to higher estimates of R_0 (but not necessarily R_e), and higher estimates of severity. In the models using GP ILI data, this manifests itself in a higher propensity to consult with a GP and in the models that use data on severe illness, it results in higher estimates of the ratio of ICU or hospital admissions per infection.
2. Induces lower estimates of attack rates, the cumulative number of infections caused by the epidemic (despite higher R_0).

3. Affects the timing of the estimated peak in the number of underlying number of infections. Specifically, prior to any observed peak in the relevant data, lowering the initial susceptibility produces an estimated peak in incidence that occurs earlier.
4. Makes little difference to the estimated pattern of GP consultations/ICU admissions etc. These are directly identified by the data and therefore are strongly anchored.

These findings are most clearly illustrated when looking at the Severity/ICU model. The SPC model, relying on direct data on GP consultations, has these sensitivities dampened by the presence of the background ILI consultations which can adapt to absorb some of the effects of changing the susceptibility parameter. Similarly, the multiplicity of data in the synthesis model and the competing strains in the SS model make these effects more difficult to clearly depict, though they are present.

Figure S10 shows some parameter correlations obtained under the SS model using data up to the end of Week 1, 2018. Each plot shows the correlation of strain-specific parameters with the initial susceptibility. For susceptibilities >0.1, there is a clear negative correlation between R_0 and π while there is no such obvious relationship for the effective reproductive number, R_e. Moving to an analysis that covers the full epidemic period, up to Week 12, based on the Severity/ICU model, Figure S11 shows a similar relationship. Here, this plot shows the posterior distributions of (top row) the susceptibility parameter π and the data dispersion parameter, η (middle row) the severity parameter, p_{ICU}, and the school holiday effect on transmission, κ (bottom row) reproductive numbers R_0 and R_e, for each of the two priors placed on the susceptibility. The top left panel in each half shows that little is learned about the susceptibility parameter, whilst there are significant shifts in the parameters p_{ICU} and R_0 between the two halves.

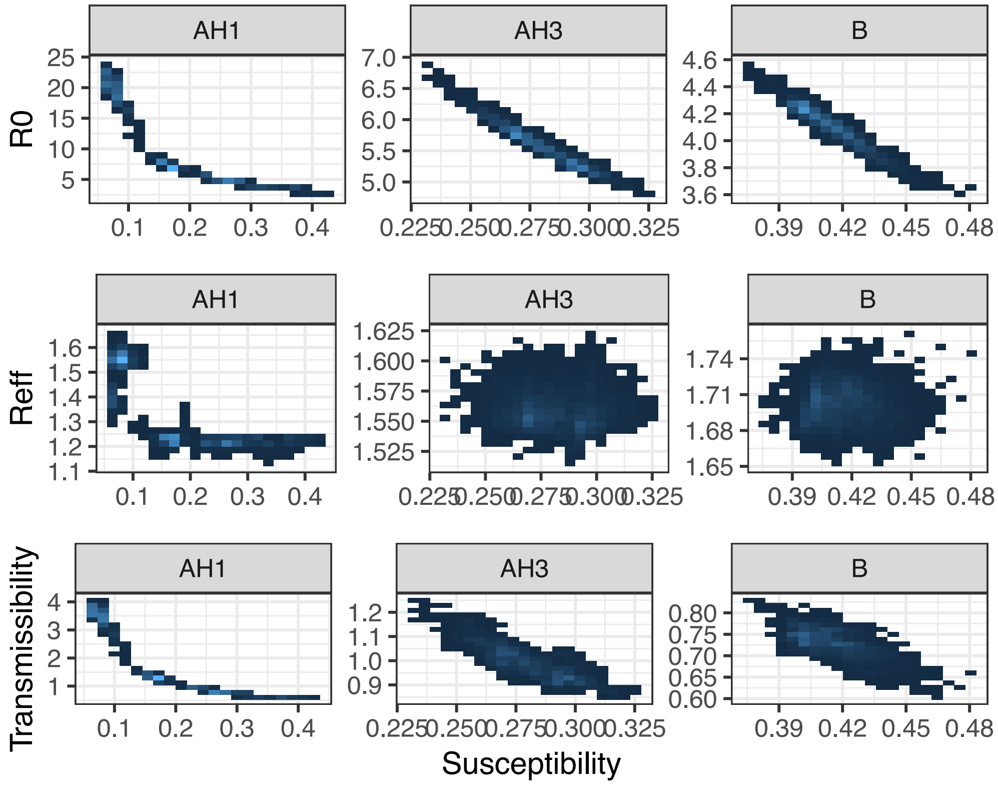

Figure S10 Posterior parameter correlations obtained from the SS analysing data up to Week 1, 2018.

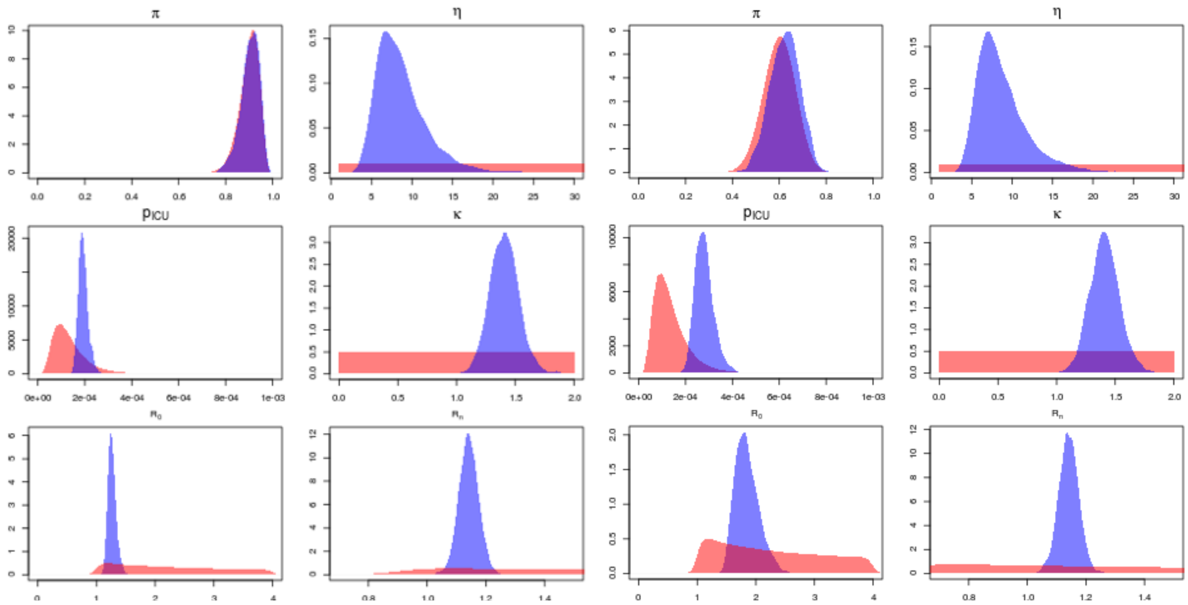

Figure S11 (On the left) Posterior distributions for parameters of interest obtained using a prior centred on π=0.9; (On the right) The same distributions obtained using a prior centered on π=0.6.

It stands to reason that, if the number of healthcare events (ICU admissions, GP consultations etc.) are anchored by data, yet there is a higher severity ratio, then it must mean there are consequently fewer infections. This also makes sense on the basis of similar values for the initial effective reproduction number R_e, but with a smaller susceptible population. The difference in the numbers infected are illustrated in the right-hand column of Figure S12 which shows the estimated cumulative numbers of infections over time. In the left-hand column of Figure S12 the plots show the estimated/forecast incidence per day. The error bars at the bottom of the plot indicate the estimated range for the timing of the peak in infection incidence. In 2018w1 (top-left panel) the estimated peak in incidence is slightly earlier when there is a lower susceptibility. The same phenomenon occurs for the analysis at 2018w2, the timing of the peak, after which the peak estimates largely coincide. In all models, prior to the peak in incidence, there is a tendency to forecast an earlier peak in incidence at the lower level of susceptibility. However, once the peak has occurred and is being retrospectively estimated, the estimated timing is independent of the initial susceptibility.

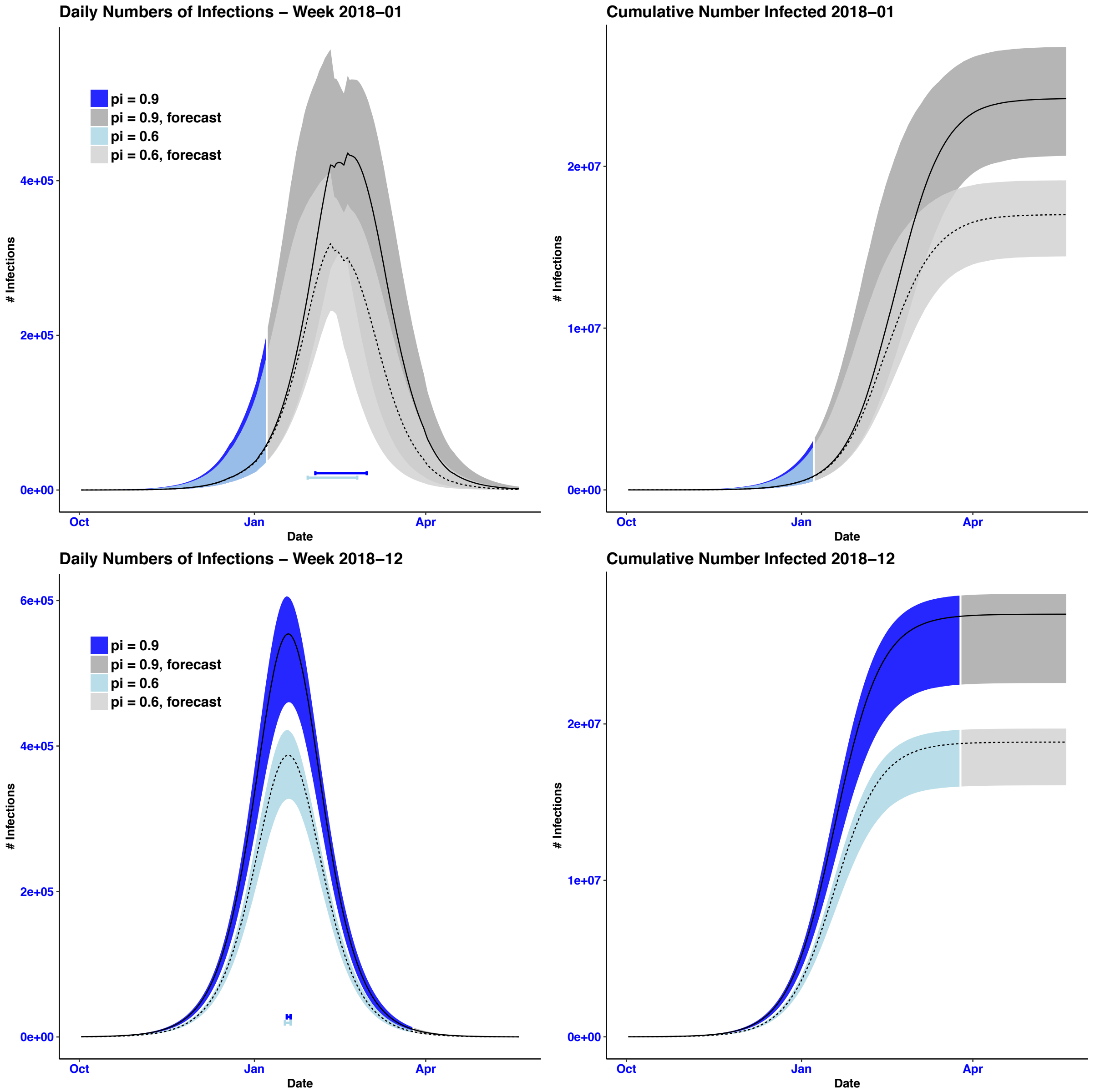

Figure S12 Left-hand column: plots of the estimated number of daily infections from the SPC model at two levels of susceptibility. The grey-shaded area corresponds to the incidence of infections forecast beyond the time of analysis (2018w1, top; 2018w12, bottom). Right-hand column: plots for cumulative incidence (and thus forecast attack rates for the influenza season).

The SPC model can provide age- and region-stratified results. The sample sizes in the serological data were too small to be able to adequately estimate age-specific susceptibilities. Whilst this didn't prove a significant barrier to regional modelling, it prevented adequate age-specific inference. If the same susceptibility is assumed across age-groups, typical contact matrices that describe the pattern of infection across the age groups such as POLYMOD (Mossong et al, 2008) drive infection towards school-age children, a phenomenon not borne out in the data. To adequately account for this, the serological data could usefully be augmented by information on vaccine uptake amongst children to build a better picture of the population's immunity profile prior to any influenza season.

This study therefore identifies the importance of having pre-season serological data analysed in a timely fashion in readiness for any coming influenza season. It enables more accurate estimation of

model parameters, can help to determine infection rates underlying the burden being placed on healthcare services and, if serological samples are representative of different geographical regions and across age groups, it can facilitate more detailed modelling at a finer granularity across the population.

References

Birrell PJ, Zhang X-S, Pebody RG, Gay NJ, and D De Angelis. (2016) Reconstructing a spatially heterogeneous epidemic: Characterising the geographic spread of 2009 A/H1N1pdm infection in England. Scientific Reports 6:29004 doi:10.1038/srep29004.

Biggerstaff, M., Cauchemez, S., Reed, C., Gambhir, M. & Finelli, L. (2014). Estimates of the reproduction number for seasonal, pandemic, and zoonotic influenza: A systematic review of the literature. BMC Infect Dis, 14.

Carrat, F., Vergu, E., Ferguson, N.M., Lemaitre, M., Cauchemez, S. & Leach, S. et al. (2008). Time Lines of Infection and Disease in Human Influenza: A Review of Volunteer Challenge Studies. American Journal of Epidemiology, 167, 775–785.

Corbella A, Zhang X-S, Birrell PJ, Boddington N, Pebody RG, Presanis AM and De Angelis D. 2018. Exploiting routinely collected severe case data to monitor and predict influenza outbreaks. BMC Public Health 18:790 https://doi.org/10.1186/s12889-018-5671-7

Held, L., Höhle, M., & Hofmann, M. 2005. A statistical framework for the analysis of multivariate infectious disease surveillance counts. Statistical Modelling, 5(3), 187–199. https://doi.org/10.1191/1471082X05st098oa

Fleming, D.M. & Elliot, A.J. (2008). Lessons from 40 years' surveillance of influenza in England and Wales. Epidemiology & Infection, 136, 866–875.

van Noort, S.P., Codeço, C.T., Koppeschaar, C.E., van Ranst, M., Paolotti, D. & Gomes, M.G.M. (2015). Ten-year performance of Influenzanet: ILI time series, risks, vaccine effects, and care-seeking behaviour. Epidemics, 13, 28–36.

Mossong, J., et al. Social contacts and mixing patterns relevant to the spread of infectious disease. PLoS Med 5, e74 (2008).

Spiegelhalter, D.J., Best N.G., Carlin B.P., Van Der Linde A. (2002) Bayesian measures of model complexity and fit. Journal of the Royal Statistical Society, Series B, 64(4) 583-639.

Tom BDM, van Hoek AJ, Pebody R, McMenamin J, Robertson C, Catchpole M, De Angelis D. Estimating time to onset of swine influenza symptoms after initial novel A(H1N1v) viral infection. Epidemiol Infect. 2011;139(9):1418-1424. Available from: http://www.ncbi.nlm.nih.gov/pubmed/21087539.doi:10.1017/S0950268810002566.
